# Surfactantless Electrophoretic Deposition of Nonaqueous CuO Nanoparticle Dispersions by Tailoring Their Surface Defectiveness

**DOI:** 10.1021/acs.langmuir.6c01947

**Published:** 2026-07-23

**Authors:** Anastasia Batenkova, Jan P. Kollender, Eric Hirschmann, Maciej O. Liedke, Maik Butterling, Daniele Casari, Patrik Schmutz, Lars P. H. Jeurgens

**Affiliations:** † Laboratory for Joining Technologies and Corrosion, 28501 Empa−Swiss Federal Laboratories for Materials Science and Technology, Dübendorf 8600, Switzerland; ‡ Department of Chemistry and Applied Biosciences, ETHZ−Federal Institute of Technology Zurich, Zurich 8093, Switzerland; § Institute of Radiation Physics, 28414 Helmholtz−Zentrum Dresden−Rossendorf (HZDR), Dresden 01328, Germany; ∥ Laboratory for Mechanics of Materials and Nanostructures, Empa−Swiss Federal Laboratories for Materials Science and Technology, Thun 3602, Switzerland

## Abstract

Electrophoretic deposition (EPD) is valued for its simplicity, cost-effectiveness, and ability to assemble nanoparticles (NPs) into micrometer-thick functional coatings, membranes, or interconnects at room temperature. In this work, we present a surfactant-free strategy to optimize the colloidal stability and electrophoretic mobility of CuO NPs in nonaqueous solutions by tuning their surface defectiveness and resulting space-charge region in the dry state. To this end, various commercial and in-house synthesized CuO nanopowders (with unknown synthesis, storage, and atmospheric aging histories) are subjected to mild, organic-free surface treatments, such as washing and subsequent annealing in O_2_ or O_3_. These optimized treatments yield “pristine” CuO surfaces with reduced defect densities—primarily monovacancies and vacancy clusters, as confirmed by combining X-ray photoelectron and positron annihilation lifetime spectroscopies—which in turn produce narrower and sharper surface space-charge regions, corresponding to higher effective surface potentials. The resulting ζ-potential of treated CuO NPs dispersed in ethanol exceeds > |±100| mV, enabling excellent colloidal stability and high electrophoretic mobility, even for very high NP loadings. A relatively high and stable steady-state EPD current is observed during EPD, which is postulated to originate from capacitive discharging of the electrophoretically deposited CuO NPs at the electrode surface. As such, coherent, uniform, porous CuO coatings with micrometer-scale thickness are obtained, entirely free of organic contaminants often detrimental for surface reactivity and material performance. These findings enable the development of efficient, up-scalable, and environmental-friendly EPD manufacturing routes of organic-free NP-based oxide coatings and membranes, independent of the nanopowder type and aging history.

## Graphical abstract

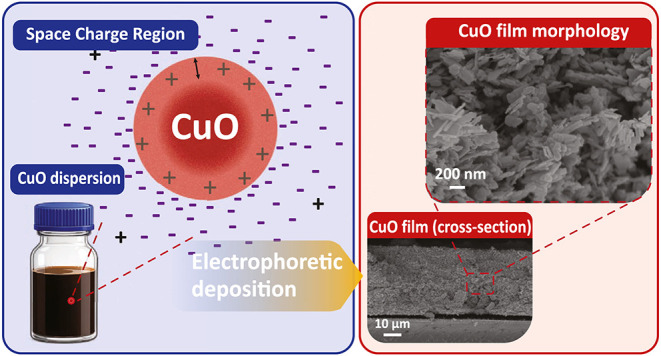

## Introduction

Electrophoretic deposition (EPD) is a technique in which charged solid particles suspended in a liquid medium migrate under an applied electric field and deposit onto an oppositely charged electrode. It is valued for its simplicity, cost-effectiveness, and ability to very rapidly assemble nanoparticles (NPs) into micrometer-thick functional films or coatings while preserving their intrinsic properties.^1,2^ Further key advantages of EPD are that it can be performed at room-temperature on (conducting) substrates of diverse geometries, including complex shapes and porous structures, and even enable patterning through electrode and template design.^3^ Various criteria must be met to ensure efficient and reproducible EPD processes from NP dispersions, in particular:

(i) **A sufficiently high NP surface charge** for electric-field-driven motion, as achieved by optimizing the so-called ζ-potential. Its value reflects the effective NP surface charge in solution and is experimentally derived from the electrophoretic mobility (μ) of the NPs in solution.^4,5^ Tuning of the ζ-potential is commonly achieved by:^3,6–10^ (a) controlled addition of stabilizing agents (e.g., surfactants or other chemical additives), (b) tuning of the dispersing medium (e.g., type of solvent, conductivity, pH, purity, temperature), and (c) tuning of the *dry* nanopowder characteristics (e.g., NP type, size, shape, concentration and surface state).
(ii) **A sufficiently high colloidal stability** of the NP dispersion to prevent NP agglomeration and coagulation during the EPD process. The colloidal stability of a NP dispersion depends on the balance between repulsive and attractive interparticle forces in solution; i.e., attractive van der Waals interactions between the NPs in solution are counteracted by repulsive forces between their electrical double layers at the NP-liquid interface.^11^ In general, absolute ζ-potential values above |20| mV indicate strong NP repulsive forces, resulting in good colloidal stability. Lower values often correlate with aggregation, sedimentation, and poor EPD film quality.^1,8^ Optimizing the colloidal stability of highly loaded NP dispersions is not only crucial for achieving efficient EPD processes, but also for improving the processability and shelf life of NP-based inks, pastes, and slurries, as applied in materials sciences, pharmaceuticals, food sciences, and cosmetics.^12–15^

(iii) **High NP loadings** (i.e., ≥5 g L^–1^) for achieving efficient and stable electrophoretic deposition rates. With increasing NP loading (and increasing temperature), Brownian motion leads to more frequent NP collisions in the NP dispersion, increasing the probability for NP aggregation, agglomeration and coagulation, resulting in less uniform, defective film morphologies.

Aqueous systems are typically favored for EPD from oxide NP dispersions, because the NP surface charge can be easily tuned by varying the pH.^16,17^ Namely, an increase/decrease of the pH with respect to the isoelectric point (IEP or point of zero charge, PZC) of the oxide phase enhances its surface charge via protonation/deprotonation, thereby allowing easy adjustment of the absolute ζ-potential. Accordingly, the colloidal stability of oxide NPs in aqueous media is typically lowest at the IEP. For example, the relatively high IEP of pH ≈ 9 for colloidal alumina suspensions^18^ results in *positive* ζ-potential values as high as +|40 ± 10| mV (at acidic pH), whereas the relatively low IEP of pH ≈ 2 for colloidal silica suspensions produces highly *negative* ζ-potentials down to about −|80 ± 10| mV (at basic pH).^19–21^ Unfortunately, depending on the type of oxide phase, acidic conditions may trigger oxide dissolution, whereas basic conditions may activate an oxide-to-hydroxide transformation,^18^ which hinders reliable determination of the “true” ζ-potential (since the chemistry and structure of the oxide NP surface alters with time).^10^ Moreover, the inevitable surface coverage of oxide surfaces by adventitious carbon species from the ambient is known to affect (i.e., generally suppress) their ζ-potential value in solution, which results in irreproducible EPD behaviors due to NP aging.^10,20^

Efficient EPD from oxide NP in aqueous dispersions (i.e., at high and stable deposition rates) is not possible under high electric fields, since water electrolyzes at the electrodes (at potential differences ≥1.23 V), producing gas bubbles (H_2_, O_2_), resulting in nonuniform defective EPD microstructures.^22^ In this case, the EPD process requires careful fine-tuning of the deposition parameters (e.g., pulsed or AC electric fields)^23,24^ and/or the use of chemical inhibitors to suppress electrolysis.^25^ To avoid such practical complications, many researchers instead opt for organic-based dispersing media, which offer much wider electrochemical stability windows for EPD.^8,26,27^ However, organic solvents—due to their lower dielectric constants and limited ion dissociation ability—typically require the use of chemical additives, such as dispersants or charging agents, to induce surface charge on the NPs, which can be combined with steric effects to add electrosteric stabilization.^3,26,28–30^ Accordingly, one or a combination of the following strategies are generally applied to optimize the colloidal stability of nonaqueous NP dispersions (see Figure 1a):

- **Surface Protonation**: Addition of acidic species (e.g., HNO_3_, AlCl_3_, I_2_) to promote protonation of surface sites, generating positively charged NPs.^31–35^

- **Surface Deprotonation**: Introduction of basic additives (e.g., organic amines) which deprotonate surface hydroxyl or acidic groups, leading to negatively charged surfaces. These bases also stabilize the release of protons by forming cationic amine species in solution.^36^
- **Adsorption**: Addition of charged polymers or surfactants to the dispersion, which can adsorb on the NP surface and confer charge while simultaneously providing steric hindrance. This electrosteric mechanism increases both electrostatic repulsion and interparticle spacing, improving colloidal stability.^26,37^

- **Prefunctionalization of the dry NP surface**: Chemical modification of the dry NP surface prior to their dispersion in the organic solvent; for example, by coating with ionic surfactants or functional molecules (e.g., silanization of silica with amine-terminated silanes) to impart a stable surface charge.^38^

**Figure 1:**
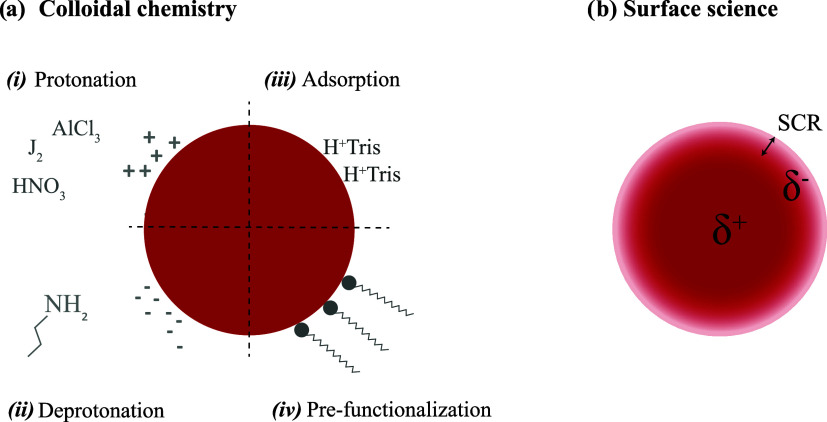
Proposed strategies for optimizing the colloidal stability of nonaqueous oxide NP dispersions through optimization of the NP surface charge (i.e., ζ-potential) to achieve an efficient and stable EPD process: (a) Schematic illustration of common stabilization mechanisms based on a **colloidal science** approach: (*i*) Surface Protonation: acidic additives donate protons to the oxide surface, inducing a positive surface charge. (*ii*) Surface Deprotonation: basic additives abstract protons from surface groups, inducing negatively charged surfaces. (*iii*) Adsorption of charged Species: polyelectrolytes, surfactants, or ions adsorb to the NP surface, providing electrosteric stabilization. (*iv*) Prefunctionalization: the dry nanoparticles are chemically modified (e.g., via silanization) prior to their dispersion to introduce a permanent surface charge; (b) Innovative **surface science** approach for establishing a narrow space charge region at the solid nanoparticle surface by surface defect engineering of the dry nanopowder (e.g., through defined annealing treatments), as performed in the present study.

Other strategies, such as the addition of small amounts of polar solvents (e.g., water) to predominantly nonpolar media can also enhance ionization and promote NP surface charging.^35^ However, given the aforementioned EPD complications due to water electrolysis, these hybrid approaches will not be considered in this work. For applications such as heterogeneous catalysis,^39^ batteries,^40^ and nanopaste sintering,^41^ any residuals of organic additives and/or surfactants in the EPD deposit can significantly compromise its properties and functionality (e.g., surface reactivity, conductivity, sinterability). A postannealing treatment in an oxidizing environment at elevated temperatures is therefore typically applied to remove (“burn”) bulk organic residues; however, chemically bound organic adsorbates on the oxide NP surfaces are very persistent and typically remain after such oxidation treatments.^10^ Moreover, residues of harmful chemical additives may be difficult to remove upon recycling or reuse of the EPD deposit, possibly inducing environmental hazards and/or pollution.^42^ In addition, high temperatures during such post-treatments activates NP sintering, resulting in a reduction of specific surface area, which may be unwanted for the targeted application.

Our research aims to develop a cost-effective, green, room-temperature, electrochemical synthesis route of organic-free CuO/Al nanothermite nanocomposites for reactive joining technologies. To this end, in a first step, micrometer-thick nanoporous CuO coatings are prepared via EPD from CuO NP dispersions in ethanol.^8^ In a second step, these nanoporous CuO scaffolds are electrochemically infiltrated by Al from an ionic liquid.^43^ The CuO/Al interfaces in the manufactured CuO/Al nanocomposites need to be pristine, i.e., free of oxides and organic contaminants, in order to lower the activation barrier for ignition and obtain a self-propagating reaction front for the reduction of CuO by Al (forming Al_2_O_3_).^44,45^ Hence, rather than introducing chemical additives to optimize the ζ-potential of the CuO NPs for the EPD step, we tune the CuO NP surface potential in the *dry* state without the use of any chemical additives by modifying the so-called surface space charge (SSC) region, as schematically illustrated in Figure 1b. To this end, as a first step, well-defined surface treatments without any addition of chemical additives, such as washing and thermal treatments in O_2_ and O_3_ gas, were applied to different types of commercial (aged) and freshly synthesized CuO nanopowders. Next, the surface-treated CuO NPs were dispersed in ethanol, while measuring their ζ-potential and colloidal stability. The resulting CuO NPs dispersions were used to fabricate micrometer thick, nanoporous CuO coatings by surfactantless EPD. As such, the present study advances fundamental understanding on the interrelationships between the CuO NP surface charge in the dry state and its ζ-potential and colloidal stability in solution.

## Results and Discussion

### As-Received CuO Nanopowders

XRD confirms that the as-received and as-synthesized nanopowders exhibit a single bulk CuO phase (no crystalline Cu_2_O, Cu­(OH)_2_ or basic carbonates detected): see Figure 2a. Williamson-Hall (W–H) analysis of the recorded diffractograms indicates average primary crystallite sizes of 13, 50 and 28 nm for the US, SA, and Empa nanopowders, respectively. These average primary crystallite sizes are comparable with the primary NP size ranges, as estimated from the TEM micrographs: i.e., (15 ± 5), (63 ± 43) and (29 ± 12 nm) for US-NP, SA-NP, and Empa-NP, respectively. Deposition of the nanopowder dispersions on the electron-transparent TEM grid leads to a mixture of “fused” aggregates and weakly bonded agglomerates of these primary CuO NPs, as shown in Figure 2c. The shape and size distribution of the primary NPs depends on the synthesis route. Note that, while the US-NPs have a narrower size range and spherical morphology, the SA-NPs are more elongated (platelet) in shape: see Figure 2c. BET analyses yield corresponding SSAs of 30, 12.5–14.3 m^2^/g (depending on batch), and 16.5 m^2^/g for the US-NP, SA-NP, and Empa-NP, respectively. The SSA values for the commercial powders are significantly lower than the suppliers’ specifications (e.g., 29.0 m^2^/g for SA), consistent with partial agglomeration/aggregation during storage and shipping and potential batch-to-batch variability.

**Figure 2:**
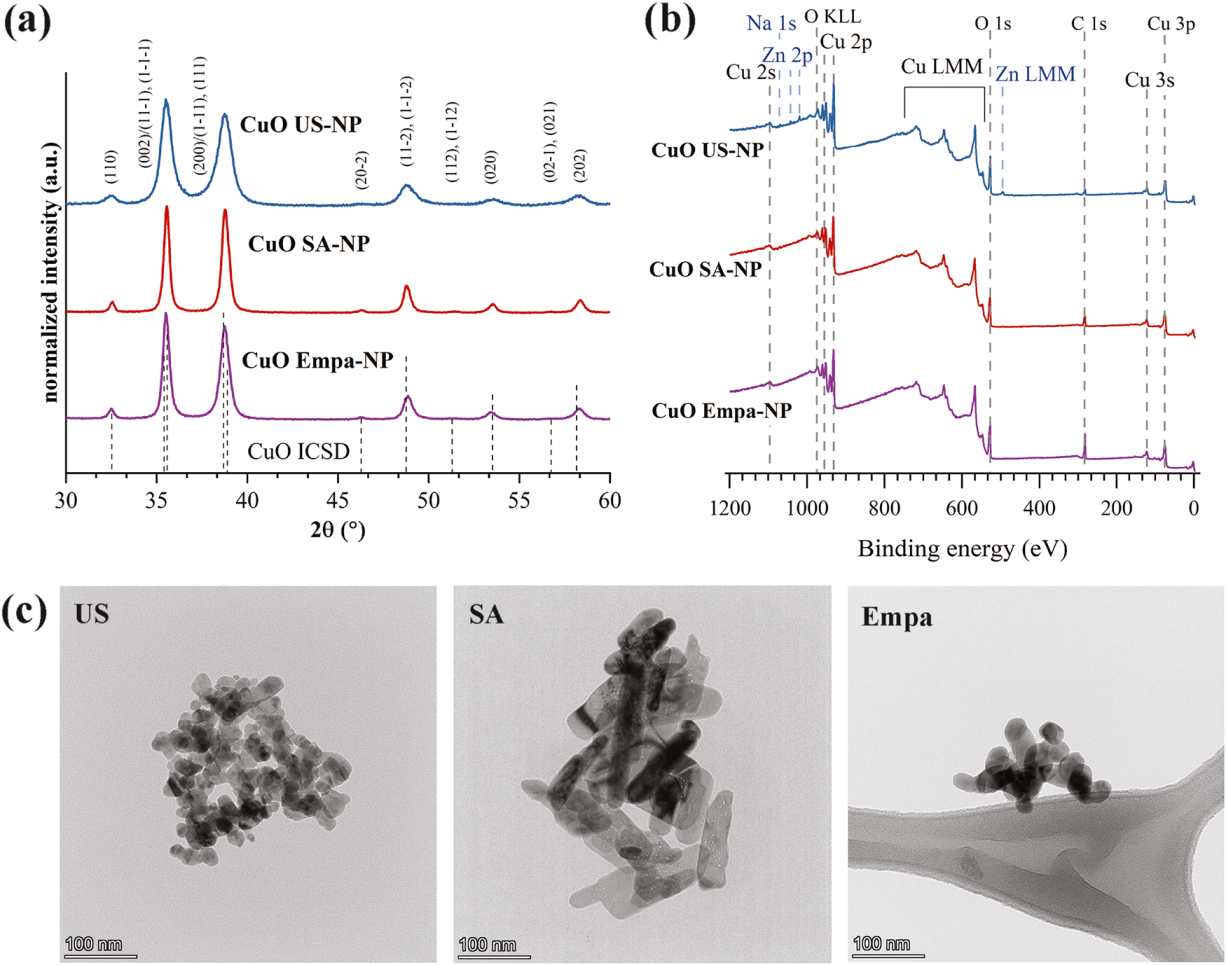
(a) XRD diffractograms and (b) measured XPS survey spectra of the as-received US-NP, SA-NP and Empa-NP CuO nanopowders (from top to the bottom). The reflections for bulk CuO (with the respective Miller indices) according to the ICSD database are indicated by the dashed lines. (c) TEM images of CuO NP agglomerates for the as-received US-NP, SA-NP and Empa-NP CuO nanopowders.

The chemical constitution of the nanopowder surfaces in the as-received state (i.e., as stored under argon and transferred without exposure to air prior to washing; see Experimental Section) was probed by XPS: see Figure 2b. All as-received nanopowders have adventitious carbon species (Adv–C) on their surfaces, as should be expected for any material exposed to the ambient.^10^ The Empa-NP powder has a significantly higher Adv–C surface concentration as compared to the commercial nanopowders, which can be attributed to additional carbonaceous residues originating from the respective wet-chemical synthesis route.^7^ Strikingly, the survey spectrum for the US-NP sample indicates residual surface contaminants, notably Na and Zn, alongside Adv-C species: see Figure 2b. After the defined washing procedure (see Experimental Section), the Na signal is largely removed, whereas a weak but persistent Zn contribution remains, suggesting chemical bonding and/or structural incorporation into the CuO US-NPs. Indeed, the certificate of the supplier of US-NP reports a bulk Zn impurity content of 195 ppm Zn.

### Effect of Surfactantless NP Surface Treatment on ζ-Potential

As demonstrated in our previous work for the commercial **SA-NP** nanopowder, atmospheric aging proceeds very rapidly for the dry CuO nanopowder, but is not observed for the corresponding CuO thin films.^10^ Such aging is accompanied by a significant drop of the ζ-potential of the (aged) CuO nanoparticles (NPs) in ethanol, resulting in both colloidal destabilization and insufficient electrophoretic mobility upon EPD. Hence, the ζ-potential correlates strongly with the structural and electronic heterogeneity of the CuO NP surface, which can be counteracted by defined surface treatments, such as washing (**as-washed**), annealing in synthetic air at 300 °C (**O**_**2**_ **@300**°**C**), annealing in O_3_ at 300 °C (**O**_**3**_ **@300**°**C**), and ozonation at RT (**O**_**3**_ **@RT**). In Figure 3a, the measured ζ-potential values for the as-received, freshly synthesized and surface-treated CuO NPs dispersed in ethanol (for concentrations in the range of 0.5–0.7.%) are compared for three different types of CuO nanopowders (i.e., two commercial ones and one in-house synthesized). These values can be compared to the reported ζ-potentials for different kinds of CuO NPs dispersed in water or ethanol (with or without additives), as tabulated in Table 1. Please note that the term “as-received” for the two commercial nanopowders implies an unknown storage/aging history and is therefore further referred to as “aged”. The CuO Empa–NP has been freshly synthesized in-house and is therefore designated as “as-washed”. The final synthesis step of the CuO Empa–NP involves air annealing at 400 °C for 4 h, which exceeds the temperature of our default annealing treatments in O_2_ and O_3_ of 300 °C. Hence, no additional surface treatments at 300 °C were performed for the Empa-NP powder.

**Figure 3:**
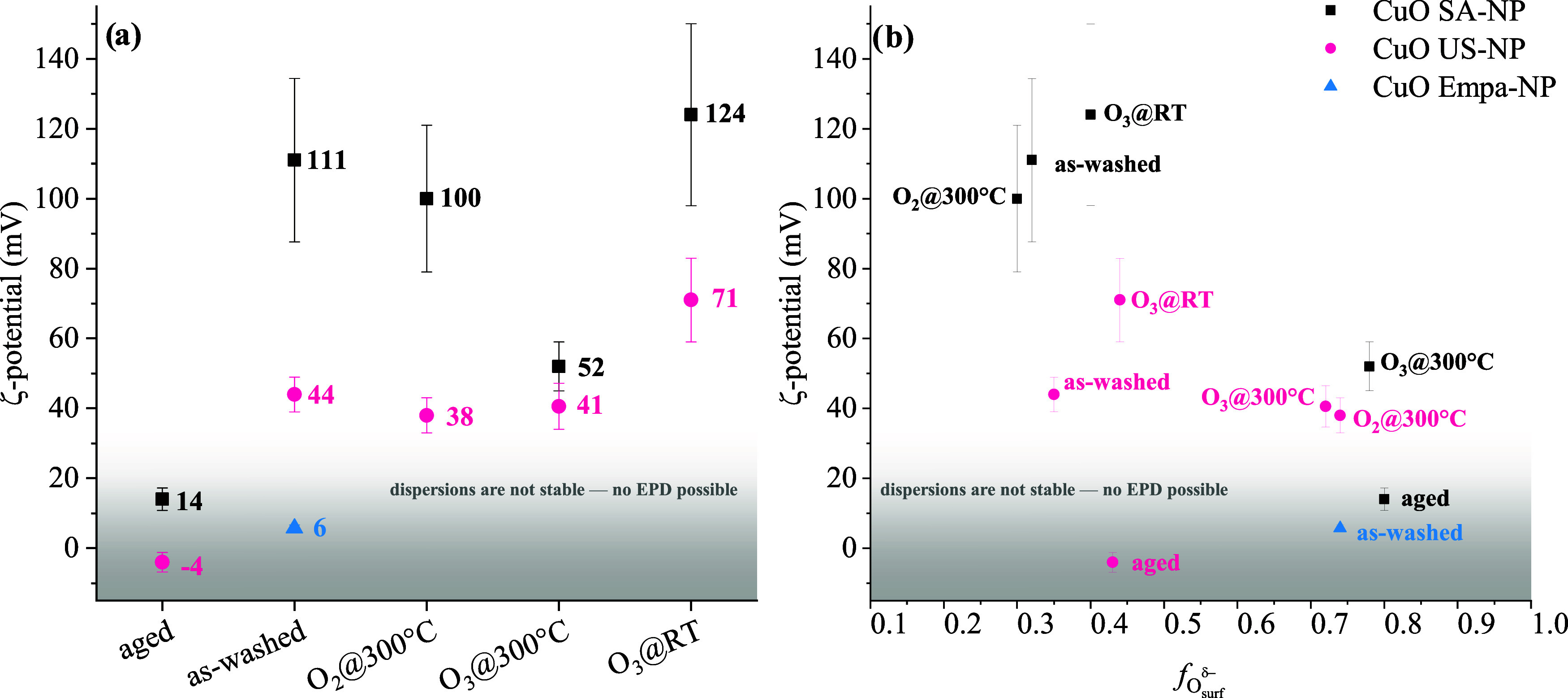
Measured ζ-potentials of aged, freshly synthesized, and surface-treated CuO nanoparticles dispersed in ethanol (0.5–0.7.%) for three types of NPs (SA–NP, US–NP, and Empa–NP) plotted versus (a) surface treatment (i.e., as-washed, O_2_@300°C, O_3_@300°C, and O_3_@RT), and (b) respective fraction of defective oxygen (sub)­surface species, *f*_O^δ−^surf_. The fraction *f*_O_surf_^δ−^_ corresponds to the area ratio of the resolved surface (O_surf_^δ−^) to bulk (O_bulk_) O 1s components by XPS:^10^ see Figure 4. The different surface treatments are described in the Experimental Section. The gray band marks the EPD-inactive region (|ζ| ≲ 20 mV). Commercial “as-received” powders have unknown aging histories (“aged”). The Empa–NP was freshly synthesized (“as-washed”), ending with air annealing at 400 °C, and therefore received no additional 300 °C treatments.

**Table 1:** Measured ζ-Potentials of Different Kinds of CuO NPs Dispersed in Water or Ethanol (with or without Additives), as Reported in the Literature^a^

media	ζ-potential (mV)	surfactant/dispersant	reference
Water	–59.8	+	^46^
Water	–48.8	-	^47^
Water	–34.6	-	^48^
Water	–30.5	-	^49^
Water	–13.7	+	^50^
Water	–1.6	-	^51^
Water	63.2	-	^52^
Water	80.0	+	^53^
Ethanol	4.4	+	^43^
Ethanol	27.9	-	^54^
Not reported	–16.0	-	^55^
Not reported	–27.3	-	^56^
Not reported	46.6	+	^57^
Not reported	63.2	-	^58^

a Please refer to the listed references for further experimental details.

As follows from Figure 3a, the commercial nanopowders have experienced atmospheric aging, resulting in very similar ζ-potential values <| ± 20| mV, below the threshold where EPD can still be performed (corresponding to the grayed area in Figure 3). The freshly synthesized Empa-NP also has a very low ζ-potential below the EPD threshold, as discussed in more detail below. After the above-mentioned surface treatments, the ζ-potential values exceed the EPD threshold only for the commercial nanopowders (i.e., US-NP and SA-NP).

CuO SA-NP: The highest ζ-potentials are obtained for the treated SA-NP nanopowder with values exceeding >| ± 100| mV for the as-washed and O_3_@RT states, which can be compared to the reported literature values for CuO NP dispersions in water or ethanol (with or without surfactant) in Table 1. Most of the literature values are also negative, although positive ζ-potentials were obtained by adding a surfactant or dispersant. The (absolute) ζ-potentials in Table 1 are all significantly lower than the highest (absolute) ζ-potentials of >| ± 100| mV, as obtained for the as-washed and O_3_@RT nanopowders in the present study. This underlines the novelty and effectiveness of our surfactantless surface treatments for tuning the NP surface charge in the dry state (see next section for a description of the underlying fundamentals).

The O_3_@300°C-treated SA-NP powder yields a considerably lower ζ-potential of around 52 mV, as compared to all other surface treatments for the same SA-NP nanopowder. To understand this anomaly, we analyzed the surface state of the O_3_@300°C-treated SA-NP by XPS: see Figure 4. Hydroxylated CuO NP surfaces readily react with organic trace gases from the ambient (e.g., aliphatic hydrocarbons, volatile organic and carbon monoxide),^59^ forming so-called Adv–C surface species, which include aliphatic alkyl-type C–C/C–H, aliphatic organic-oxygen-type (e.g., C–OH, C–O–C, C=O, O–C=O) and to a much lesser extent carbonate-type (CO_3_) surface species.^10^ In particular, the formation of organic-O surface species disturbs the stoichiometry and crystallinity of the pristine CuO NP surface, as evidenced by the coinciding appearance of defective oxygen species in the measured O 1s XPS spectra (further denoted as O_surf_^δ−^): see Figure 4. The respective Cu 2p_3/2_ main peak also has a larger FWHM of about 3.4 eV with a less pronounced splitting of its associated satellite (see Figure S4), indicative of a distortion of [CuO_6_] nearest-neighbor coordination spheres in CuO.^10,60^ In Figure 3b, the measured ζ-potential is plotted as a function of the surface fraction of such defective O surface species, *f*_O^δ−^surf_ (with respect to the CuO NP bulk lattice^10^). It follows that the relatively high surface fractions, *f*_O^δ−^surf_, for the aged and O_3_@300°C SA-NPs indeed correlate with a relatively low ζ-potential: see *black squares* in Figure 3b. This suggests that the coformation of organic-O species and defective O_surf_^δ−^ subsurface species not only occurs during atmospheric aging, but also upon exposure to ozone at elevated temperatures of 300 °C. In other words, both hydroxyl surface groups and gaseous ozone species seem to promote coformation of organic-O species and defective O_surf_^δ−^ subsurface species. Since the O_3_@300°C treatments are carried out at an elevated temperature and for high ozone concentrations, such O_3_-mediated surface reactions will be promoted. This also rationalizes the higher ζ-potential of the O_3_@RT-treated SA-NP powder (as compared to the O_3_@300°C state; see Figure 3), since ozonation at room temperature and at lower ozone concentrations mainly acts on the outer CuO-NP surface (without thermally activated atomic rearrangements of mobile carriers/defects in the subsurface region below).

**Figure 4:**
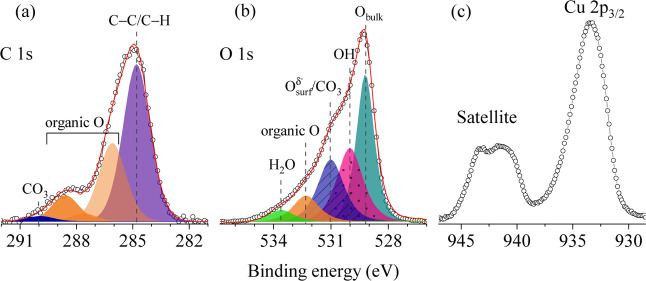
Measured and reconstructed XPS spectra of the (a) C 1s region, (b) the O 1s region, and (c) the Cu 2p_3/2_ region, for the aged CuO SA-NP after annealing at 300 °C in O_3_-flow (denoted as O_3_@300°C). The different chemical species, as resolved by constrained peak fitting of the C 1s and O 1s regions, are represented by specific colors with the different types of organic-O species being combined in an orange color scale. The imposed constraints for the chemical shifts and FWHM’s of the fitted synthetic main peaks are reported in ref 10. The C 1s and O 1s spectra shown were normalized by the integrated intensity of the Shirley background corrected spectral envelope to correct for variations in absolute photoelectron intensity, depending on the selected position and area for XPS analysis. The Cu 2p_3/2_ spectra is not normalized, reflecting the peak position and FWHM of the main peak and the shape of the respective satellite.

CuO US-NP: The positive ζ-potential values for the CuO US-NP also increase after the aforementioned surface treatments, but all remain well below those of the SA-NP (see *pink circles* in Figure 3a). Nevertheless, the measured ζ-potential values in the range of |40–60| mV for the treated US-NPs still fall into the category of highest (absolute) ζ-potentials reported in the literature (see Table 1), being sufficiently high to obtain good colloidal stability for an efficient EPD process. XPS analysis of the as-washed CuO US-NP reveal persistent Zn bulk impurities (in accord with the supplier sheet): see Figure S3.1. Unlike adventitious carbon surface species that can be partially removed and/or reduced through annealing in O_2_ and O_3_,^10^ Zn is incorporated into the CuO lattice due to the near-identical ionic radii of Cu^2+^ (0.73 Å) and Zn^2+^ (0.74 Å).^61,62^ Once embedded, these foreign bulk dopants cannot be removed by such mild surface treatments and therefore introduce permanent structural defects that alter the electronic structure^10^ and thereby the effective surface charge, leading to a smearing of the SSC region (as discussed in the next section). These Zn bulk impurities thus rationalize the consistently lower ζ-potential as compared to the CuO SA-NP.

CuO Empa-NP: Finally, the ζ-potential of the freshly synthesized Empa-NP powder of about 6 mV could not be enhanced beyond the EPD threshold. Indeed, EPD of the Empa-NP dispersions was attempted, but failed (i.e., no deposition occurred), even when applying EPD potentials as high as 15–100 V. This aligns with our previous work, showing that EPD of the Empa-NPs from ethanol dispersions can only be achieved by adding a surfactant (e.g., 0.5 wt % acetylacetone).^8^ Although the freshly synthesized CuO Empa-NP falls in the “as-washed” category, it could also be tentatively assigned to the “annealed” surface states, since it was obtained by calcination of a malachite precursor phase at 400 °C.^7^ XPS analysis of the resulting CuO Empa-NP in Figure S2.3 shows that the Cu 2p_3/2_ region exhibits a distorted satellite structure with a relatively broad FWHM of the respective main peak similar to that of the “aged” nanopowders. Consistently, the surface fraction of defective-O surface species is relatively high (resulting in a low ζ-potential): see *blue triangles* in Figure 3b. This outcome may be expected, given that the synthesis proceeds via calcination of a malachite precursor phase and therefore remnants of the malachite precursor might persist within the lattice, modifying the electronic structure and surface composition from that of pure, crystalline CuO. Even more aggressive postsynthesis surface treatments, such as annealing in ozone flow, did not produce a pristine-like CuO surface state (see Figure S2.3). Annealing at even higher temperatures would induce NP growth and nanopowder sintering, which is undesirable.

### Correlation between NP Surface Charge and ζ-Potential

Localized space charge regions are an intrinsic property of practically all oxide surfaces, interfaces, as well as grain boundaries.^63,64^ The oxide NP surface space charge (SSC) region corresponds to the surface region where defect and carrier concentrations deviate from the bulk (i.e., the local charge density is nonzero). The resulting net surface charge is screened by the redistribution of mobile carriers and/or defects (e.g., vacancies, dopants) in the bulk to preserve overall charge neutrality.^64^ The SSC thus produces a build-in electrostatic potential at the outer surface (i.e., at *x* = 0) relative to the bulk (i.e., at *x* = ∞). The corresponding potential gradient with increasing depth, *x*, below the surface gives rise to a build-in electric field, which bends the conduction and valence bands in the SSC region, as illustrated for CuO in Figure 5a.

**Figure 5:**
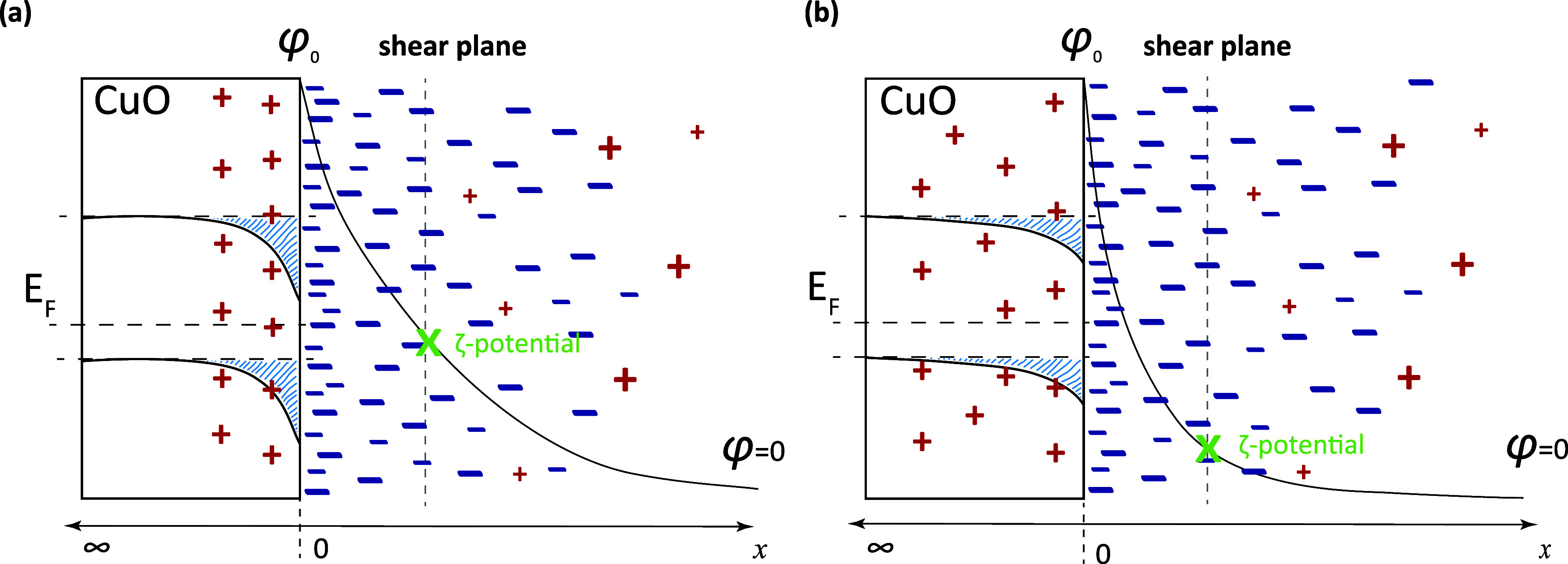
Sketch illustrating the correlation between the surface space charge region (SCR) in CuO and its corresponding ζ-potential in solution: (a) for the case of a “pristine” (i.e., crystalline and clean) CuO surface, and (b) for the case of a more defective CuO surface, such as those resulting from atmospheric aging, bulk impurity dopants (e.g., Zn) and/or ozone treatments above room temperature. *E*_F_ denotes the Fermi level. The SCR produces a build-in electrostatic potential, φ_0_, at the outer surface (i.e., at *x* = 0) relative to the bulk (i.e., at *x* = ∞). The corresponding potential gradient with increasing depth gives rise to a build-in electric field, which bends the conduction and valence bands in the SSC region. The measured ζ-potential at the oxide/liquid interface is related to the potential drop in the electrical double layer (EDL), as established to screen the build-in electrostatic surface potential. The ζ-potential is defined at the shear plane where the liquid velocity relative to the NP becomes zero. The EDL in solution has to screen a higher build-in surface potential if the SSC region at the oxide surface is more narrow and concentrated at the outer surface, as is the case in (a) compared to (b).

Tailoring of space charge regions by impurity doping is a well-established engineering concept in semiconductor physics; for example, in *p*–*n* junctions for microelectronics and solar cells, where the functional space charge region (SCR) at a *p*–*n* junction is commonly denoted as depletion region (i.e., the mobile carrier densities, i.e., electrons or holes, are zero in the depletion region).^65^ Ionic and partially ionic compounds, like oxides (with mixed ionic–covalent bonding character^64^), have a limited mobility of charged species (e.g., ions, vacancies, Interstitials, electrons, holes) that strongly depends on e.g., crystallinity, defects, doping, stoichiometry, and temperature. Such ionic compounds are therefore associated with “kinetically-frozen” SSC regions and resulting “build-in” surface potentials in the solid state, which—in the absence of doping—typically originate from defective nonstoichiometric transition regions.^66^ For example, the space charge region in Al/Al_2_O_3_ tunnel junctions in microelectronics is constituted by excess Al cations at the Al/Al_2_O_3_ interface, which introduce donor levels in the band gap, acting as an electric potential energy barrier for electron tunneling.^67^ For the solid–liquid interface between an oxide phase and a liquid electrolyte (*here*: CuO/ethanol), the build-in electrostatic potential at the oxide surface will be screened by mobile ionic species in the EDL, thus establishing a potential drop in the EDL which dictates the measured ζ-potential (as defined at the shear plane where the liquid velocity relative to the NP becomes zero^4,5^ ): see Figure 5a.

For pristine (i.e., clean and well-crystalline) oxide surfaces, the SSC region typically originates from a reconstruction of the oxide surface to compensate for surface polarity, thereby lowering the surface energy.^64,68–70^ For example, anhydrous oxide surfaces are known to reconstruct by hydrolysis of metal–oxygen surface bonds (i.e., by surface hydroxylation).^18,70^ These surface-reconstruction-induced SSC regions critically depend on the crystallographic orientation and chemical termination of the surface plane (e.g., the degree of hydroxylation, segregation and the presence of adsorbed surface species),^68,70–73^ being much more pronounced for oxide NPs as compared to thin oxide films.^10^ The corresponding SSC region for such reconstructed or surface-terminated oxides (which may include specific surfactants; see Figure 1) is very narrow and sharp (i.e., confined at the outer surface), which gives rise to a relatively high build-in surface potential and thereby a relatively high ζ-potential in solution: see Figure 5a. Notably, much higher effective surface potentials can be achieved by a narrow surface space charge region at a pristine oxide surface as compared to conventional strategies targeting a dense coverage of the NP surface by charged (or polar) molecular species: i.e., the spatial distribution of relatively large charged molecular surface species on the NP surface will generally be lower than the surface charge density achieved by a localized SSC region at the reconstructed NP oxide surface. Based on the tendency of vacuum-reconstructed CuO surfaces to form Cu-rich, oxygen-deficient sites and donor-like oxygen vacancies,^74,75^ it may be speculated that pristine CuO NP surfaces exhibit a positive effective surface charge (i.e., a positive build-in electrostatic potential, φ_0_, at the outer surface; see Figure 5a), which complies with a positive ζ-potential in ethanol solution. In this regard, it should be emphasized that oxides which are constituted of a multivalent cation, such as Cu (1+ and 2+), Mn (2+, 3+ and 4+) and Fe (2+ and 3+) can be much more easily tuned by dedicated surface treatments to exhibit defective nonstoichiometric SSC regions at their outer surface. Oxides such as SiO_2_ and Al_2_O_3_ strive to maintain a single valence state of the cation constituent in the oxide (i.e., 4+ for Si and 3+ for Al) and therefore tend to more strictly obey their stoichiometric composition; hence, it will be more difficult to induce nonstoichiometric (defective) SSC regions by dedicated surface treatments.

The formation of organic-oxygen species on the NP oxide surface during atmospheric aging (or mediated by interaction of ozone during post-treatment) is accompanied by the creation of additional defective oxygen species in the NP subsurface region and an associated distortion of defined [CuO_6_] nearest-neighbor coordination sphere in CuO.^10^ This experimental observation is in line with theoretical predictions stating that removal of O atoms from CuO(111) surfaces by reduction (e.g., by reaction with reducing adventitious-C species during aging) results in thermodynamically preferred formation of O vacancies in the CuO subsurface region.^74^ Consequently, the initially sharp and well-defined SSC region of the pristine CuO surface is modified: i.e., a redistribution (spread) of net surface charge from the outer surface toward the subsurface occurs, which results in a broadening of the SSC region, as illustrated in Figure 5b. Quantitative XPS analysis of nanopowders is extremely challenging, since the photoelectron signals ratio originating from surface and core regions of the NP assembly vary from point to point.^10^ Therefore, the average width of the SSC region deduced from the defective O 1s species by XPS cannot be quantified, but only estimated to be of the same order of magnitude as the XPS probing depth (of about 6 nm^10^). The extent to which a broadening of the SSC region (upon aging) affects the effective surface potential (i.e., the build-in electrostatic potential, φ_0_, at the outer surface) depends on the Debye screening length (λ_D_) in CuO, i.e.

1 $$\lambda_{D}=\sqrt{\frac{\varepsilon_{0}\varepsilon_{\mathrm{CuO}}k_{B}T}{ne^{2}}}$$

where ε_CuO_ is the relative dielectric constant of CuO, *n* the free carrier concentration in CuO, *T* the temperature, *e* the elementary charge, ε_0_ the permittivity of free space (≈8.854 × 10^–12^ C^2^ N^–1^ m^–2^), and *k*_B_ Boltzmann constant. Very rough estimates for λ_D_ vary in the range from few to tenths of nanometers, depending on the adopted values for *n* and ε_CuO_. For example, for pure CuO with a pristine surface state, it may be speculated that ε_CuO_ ∼ 18 and *n* ∼ 10^17^–10^18^ cm^–3^ (note: this is a very rough estimate based on the very large spread of reported literature values for *n* and we therefore refrain from citing any specific paper). These approximate values for ε_CuO_ and *n* result in an estimated Debye length in CuO in the range of λ_D_ ∼ 5–15 nm, which is of the same order of magnitude as the probing depth of our XPS analysis (of about 6 nm^10^).

It may be questioned if the surface charge of a pristine CuO NP dispersed in ethanol is not also governed by protonation/deprotonation of the CuO surface by trace water impurities (note: the effect of ionic impurities in ethanol can be neglected, as confirmed by XPS). The isoelectric point of CuO films and CuO NPs is typically higher than pH 8–9, which would suggest a slightly positive surface charge of CuO in ethanol due to protonation/deprotonation of the CuO surface by trace water. Such weak protonation of the CuO NP surface by itself cannot rationalize a positive ζ-potential exceeding +100 mV, but may add up to the positive surface potential of the CuO NP itself.

For atmospheric aging of pristine CuO NP surfaces, one must distinguish between neutral hydrocarbon carbon and oxygenated organic-C surface adsorbates.^10^ Aliphatic adv-C surface species do not add fixed surface charge (e.g., strong dipoles) to the CuO NP surface but rather passivate active Cu/O surface sites, thus acting as a barrier, shielding the effective surface potential of the CuO NPs experienced by the liquid. So aliphatic adv-C on the pristine CuO NP surface can reduce the measured ζ-potential even without strongly changing the intrinsic SSC region of the CuO NP underneath. Organic-O groups such as C–OH, C–O–C, C=O, O–C­(=O), and carbonate-like species create much stronger chemical bonds with the CuO NP surface, which introduces dipoles that effectively shift negative charge density toward the outer NP surface. Consequently, organic-O adsorbates modify the CuO SSC region; i.e., the SSC region becomes chemically coupled to the organic-O adsorbates. Indeed, XPS analysis evidence that atmospheric aging generates (immobile) O vacancies toward the subsurface region, which suppresses the mobile carrier concentration (thus extending the Debye length). Accordingly, it is concluded that the effective surface potential at the CuO NP surface (and thereby its ζ-potential in solution) is suppressed upon atmospheric aging due to a broadening and more heterogeneous nature of the SSC region (with SSC width ≤ λ_D_ in CuO).

Malachite-like phases formed during aging or residing as remnants from the synthesis route (e.g., for the Empa-NP), disrupt the purity and crystallinity of the CuO NP surface (see above), introducing a disordered (partially amorphous) and broad SSC region. Consequently, both the built-in surface potential as well as the corresponding ζ-potential in solution are reduced. Copper carbonates formed on Cu^2+^ surfaces also deteriorate the catalytic efficiency of copper, as attributed due to a reduced mobility of charged species.^76^ Similarly, foreign impurities, such Zn for the US-NP nanopowder, broaden the SSC region, resulting in a lower effective surface potential and a lower ζ-potential. It is thus concluded that mild and organic-free surface treatments, which restore the purity and crystallinity of the CuO NP surface (i.e., washing, O_2_@300°C, and O_3_@RT; as evidenced by the XPS analysis both in terms of surface composition and surface defectiveness; see Figure 3), effectively sharpen the SSC, thereby increasing both the built-in surface potential and the measured ζ-potential in solution.

### Electrophoretic Deposition (EPD) from CuO NP Dispersions

This section addresses the optimized process parameters and resulting film microstructures for EPD from the surface-treated CuO NPs dispersed in ethanol. The discussion centers on the SA-NPs, since they possess the highest ζ-potential after the different surface treatments and do not contain foreign impurities (like Zn for the US-NP). Corresponding results for the treated US-NP nanopowder are included in the Supporting Information (cf. 1:Figure S5.2). EPD was performed on conductive FTO-coated glass for a fixed CuO NP concentration of 5 g L^–1^ (0.6 wt.%). Lower CuO NP concentrations resulted in inefficient EPD deposition rates and incoherent (or no) film formation. Because the concentrated CuO NP dispersions were fully opaque, the resulting aggregate/agglomerate size distributions in dispersion could not be assessed by conventional DLS (Dynamic Light Scattering) method, as in e.g., ref 7. Although sufficient dilution would enable DLS measurements, the resulting particle interactions would differ from those in the highly loaded dispersions used for EPD, where electrical double-layers overlap and concentration-dependent many-body particle–particle interactions become relevant: see Section S9.1 of the Supporting Information. Hence, the ζ-potential obtained from diluted CuO dispersions will not be representative of the ζ‑potential in the highly loaded dispersions used for EPD.^8^ In this regard, it is important to note that, as a first step, all the CuO nanopowders were successively washed in ethanol and water for three times, which removes the majority of ionic surface contamination. The surface chemistry of the as-washed and surface-treated CuO NPs was carefully monitored by XPS before dispersing them in ethanol, as well as after EPD. No foreign impurities nor ionic contaminants were detected on these surfaces by XPS (besides from Zn bulk impurities and some residual Mg). While we cannot fully exclude contributions from solvent-specific charging or trace water impurities, surface contamination control by XPS for each processing step indicate that the dominant factor governing the ζ-potential in the present study, is the CuO NP surface state (i.e., in the absence of bulk impurities like Zn), rather than uncontrolled ionic contamination of the ethanol medium.

CuO film deposition was performed at a DC voltage of 25 V applied on the FTO-glass for deposition times in the range of 7–14 min, depending on the targeted film thickness: see Figure 6 and Table 3 (*note*: a lower deposition voltage of 15 V was found to be optimal for EPD with the O_3_@300°C-treated CuO US-NP). These optimized EPD process parameters resulted in CuO films with reproducible morphologies and uniform thicknesses. Nonpristine (i.e., contaminated or aged) CuO NP surfaces typically result in no or highly incoherent EPD film formation. XPS monitoring of the surface state of the freshly treated CuO NPs prior to the preparation of a NP dispersion (e.g., by quick and easy Cu 2p_3/2_ satellite-shape fingerprinting^10^), turned out very useful and effective to prevent irreproducible EPD runs. The occurrence of a transient current density >10 μA/cm^2^ at the onset of the EPD process also served as a practical indicator for unsuccessful EPD runs, which probably arises from a too low ζ-potential and colloidal instability. In nonaqueous systems, such high transient current densities at the onset of the EPD process generally originate from redox processes involving charging agents and/or electrolyte contaminants. Admittedly, ethanol is highly hygroscopic (i.e., it readily absorbs water from the atmosphere) and therefore a high transient current density at the onset of the EPD process may also originate from electrolysis of residual water. However, for the EPD from CuO NPs dispersed in ethanol without surfactants, as performed in this study, the EPD process is practically indistinguishable (by the monitored current density) when using freshly opened absolute ethanol or ethanol stored for six months in a lab environment. Moreover, a control experiment performed with plain ethanol (without CuO NPs) yields a markedly lower current density of ≈4 μA/cm^2^ (see S7.1 in Supporting Information). This indicates that the presence of CuO NPs accounts for more than half of the current density observed during EPD.

**Figure 6:**
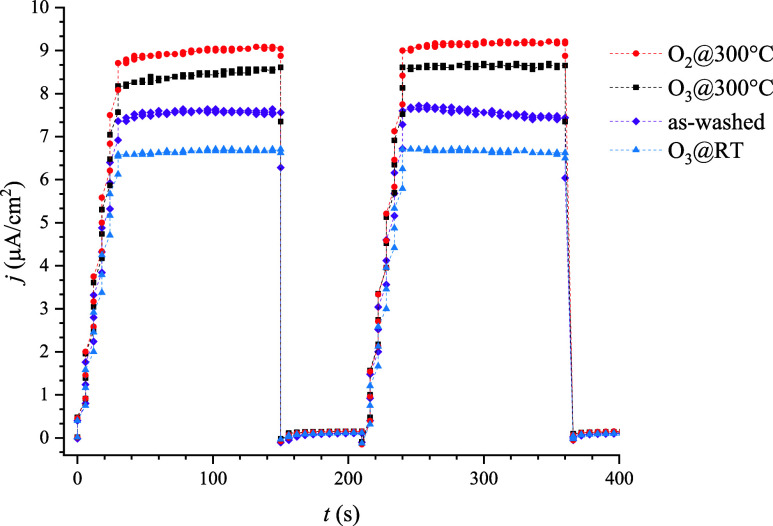
Current density versus deposition time for the electrophoretic deposition from the CuO SA-NP dispersions (in ethanol) at a fixed NP concentration of 5 g L^–1^, applying a DC voltage of 25 V. The EPD parameters correspond to those reported in Table 3. The EPD profiles are shown for the following surface treatments of the SA-NP nanopowder: O_3_@RT (blue triangles), as-washed (purple diamonds), O_3_@300°C (black squares), and O_2_@300°C (red circles). Slightly different steady current densities are observed during the film deposition stage, depending on the NP surface state (and respective ζ-potential; see Figure 3).

Representative cross-sectional SEM micrographs of the CuO films obtained after four EPD cycles deposition using differently treated SA-NPs are shown in Figure 7. The SEM cross sections reveal rather uniform porous morphologies, independent of the surface treatment. The platelet-shaped morphology of the primary CuO SA-NPs, as observed by TEM (see Figure 2c), is preserved after the surface treatment and the EPD process. A SEM cross-section at lower magnification (see Figure S5.1) demonstrates laterally uniform substrate coverage with an approximately constant film thickness of ≈30 μm. Decreasing the deposition time from 14 to 7 min produces a film roughly half as thick (≈15 μm). The rather uniform pore morphologies and film thicknesses, as evidenced in Figures 7 and S5.1, are beneficial for subsequent homogeneous electrochemical infiltration by Al from an ionic liquid,^43^ as foreseen as the next step in our research (see Introduction section). For comparison, Figure S5.2 shows cross-sectional SEM micrographs of CuO films prepared from differently treated US–NPs, having a spherically shaped primary NP morphology and a narrower NP size range (see Figure 2c): consequently, denser but less-uniform film microstructures, constituted of large NP-agglomerates, are obtained. It is thus concluded that the uniformity, morphology and porosity of the EPD films are mainly determined by the shape and size distribution of the original nanopowder, being largely independent of the NP surface pretreatment.

**Figure 7:**
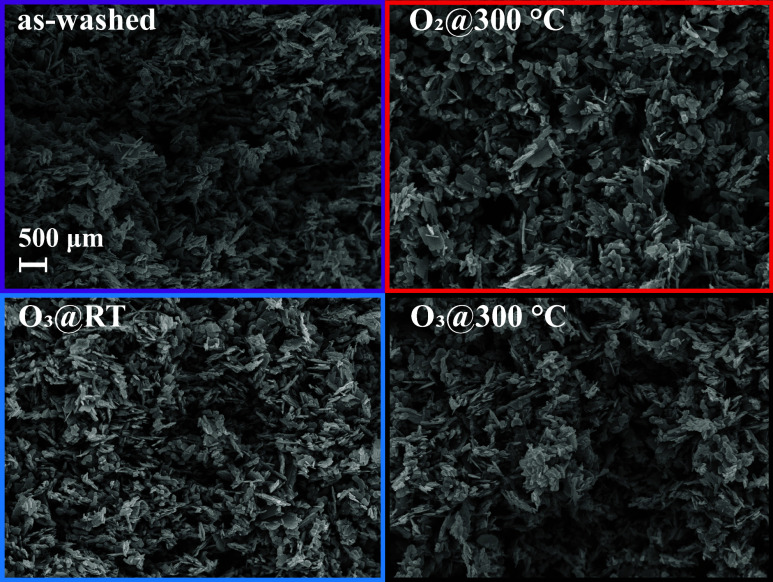
Cross-sectional SEM micrographs of the CuO films, as prepared from the SA-NP dispersions by EPD (according to profile described in Table 3), for different surface-treatments of the CuO SA-NP (i.e., as-washed, O_2_@300°C, O_3_@RT, and O_3_@300°C).

XPS analysis of the deposited CuO films only detects copper, oxygen, and adventitious carbon at their surfaces (i.e., the EPD process did not introduce any contamination): see Figure S3. Evidently, any tuned surface state of the CuO NPs would be lost if the CuO NPs are not stable in solution (e.g., slowly dissolving, as would be the case in aqueous solutions at low and high pH) and/or partially reduced at an externally applied potential of 25 V (as can be excluded based on cyclic voltametry studies of the CuO NP dispersions: see Figure S4.1). High-resolution Cu 2p_3/2_, C 1s, and O 1s spectra before and after the EPD process show no significant differences between the surface states of the dry nanopowder (i.e., after surface treatment) and that of the respective EPD film: see Figure S3.2. It is thus concluded that the tailored (pristine) surface state of the dry CuO NPs is preserved when dispersed in ethanol, as well as when applying an external DC voltage of up to 25 V during EPD. These experimental findings also comply with the observation that the surface-treated CuO SA-NP and US-NP dispersions exhibit an excellent long-term colloidal stability in ethanol (remaining stable over days upon storage under laboratory conditions).

As reflected in Figure 6, the current density during the main deposition stage is rather stable, varying only slightly across the different surface treatments (stepwise increasing in the order O_3_@RT, as-washed, O_3_@300°C to O_2_@300°C). Unlike classical electrochemical deposition, for which the measured current is predominantly Faradaic (arising from redox reactions at the electrode surfaces), EPD of oxide NPs involves little to no sustained electrode reactions (in the absence of water splitting). Still, the measured EPD currents in Figure 6 may reflect minor transient electrochemical contributions from (i) slight reduction of Cu^2+^ to Cu^+^ (see below) and/or (ii) electrolysis of residual water (see above). However, given the relatively large applied potential of *E*_EPD_ ≈ 25 V relative to the water-splitting threshold of ≈1.23 V, any residual water in ethanol is expected to be consumed within the first minutes of the EPD process. Such minor transient electrochemical reactions cannot account for the steady current observed during the main film deposition stage. As postulated below, this steady EPD current may arise from capacitive discharging of the CuO NPs at the electrode surface.

The transformation of the migrating NPs into a dense, coherent deposit is generally considered as the most complex and crucial step of the EPD process, since film formation at the electrode surface necessitates a reduction of the interparticle repulsive energy barrier by a distortion of their surrounding EDL to promote coagulation.^3,8,77^ While colloidal stability requires high repulsive interparticle forces in solution (i.e., a high ζ-potential in solution), cohesive film formation relies on attractive interparticle forces.^8^ As discussed above, the CuO NPs have an intrinsic (build-in) surface charge and therefore can be considered as a charged capacitor. Hence, once the electric-field-assisted migrating CuO NPs “hit” the electrode surface, they may exhibit a capacitative discharge, which would rationalize the stabilization of a non-Faradaic current during the main deposition stage. Upon incorporation of the deposited NPs into the thickening film, the EDL surrounding the CuO NPs in the liquid will be distorted^77^ and, consequently, the SSC region of the CuO NPs will no longer be effectively screened by the liquid EDL (see Figure 5): i.e., a realignment of their electronic structures at contacting solid–solid interfaces (i.e., CuO/CuO) is required, which may trigger such a capacitive discharge. A theoretical description and deeper understanding of the here-postulated capacitive discharging mechanism would require in-depth model calculations, which are beyond the scope of the present study. The formation of a *cohesive* CuO film by the EPD process also indicates that the repulsive interparticle forces in the liquid (by the interacting EDLs of neighboring NPs) are replaced by net attractive (Coulombic) forces between the NPs in the EPD deposit. A capacitive discharging of CuO NPs at the electrode surface may also rationalize the small but significant differences in steady EPD current density across the differently treated SA-NPs; they could originate from different defect populations in their build-in SSC region, as evidenced by positron annihilation lifetime spectroscopy (PALS) of the deposited CuO films (see what follows).

### Types of Defects in the EPD CuO Films, Evidenced by Positron Annihilation Lifetime Spectroscopy (PALS)

PALS is highly sensitive to point defects in the bulk of materials and at interfaces, and can also detect pores/voids at different depths below the surface.^78,79^ For most oxides and semiconductors, measured PALS spectra comprise multiple lifetime components associated with distinct defect classes and open-volume sites.^80,81^ The positron lifetime generally increases with defect and pore (open volume) size. Lifetimes in the picosecond range (90–500 ps) typically reflect intrinsic/extrinsic point defects (e.g., vacancies, interstitials) within the bulk and at interfaces/grain boundaries, whereas longer lifetimes in the sub- and nanosecond range are commonly assigned to orthopositronium annihilation in nanometer-sized voids. Nevertheless, unambiguous attribution to specific types of defects by PALS requires support from DFT calculations.

CuO SA-NP films with a near-uniform thickness of ≈30 μm were prepared by EPD from the CuO SA-NPs with different surface states (i.e., as-washed, O_2_@300°C, O_3_@RT, and O_3_@300°C) using the established procedure (see Table 3). Next, these EPD films were analyzed by PALS, while varying the positron implantation energy from 2 up to 18 keV (corresponding to a maximum probing depth of a few micrometers) to evaluate the defect distributions as a function of depth: see Figure S6.1. The measured spectra all exhibit a ±5–8% variation of their (normalized) signal intensities over the implantation energy range 10–18 keV (probing more bulk), which indicates a rather homogeneous defect distribution across the depth of the porous EPD films. To identify the different types of defects contributing to positron trapping, the spectra were curve-fitted using the PALSfit software,^82^ introducing a total of four decaying constants known as positron lifetimes, τ_1_ to τ_4_: see Experimental Section for more details. The deduced positron lifetimes for the different defect types identified by PALS are listed in Table S6.1. The relative intensities of each of these defect types in the EPD films of the differently surface-treated SA-NPs are gathered in Table 2.

**Table 2:**
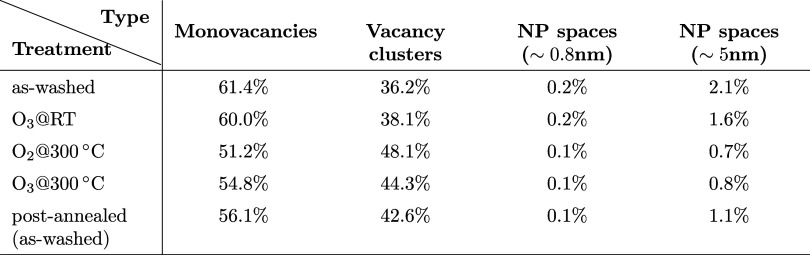
Relative Intensity (in %) of Monovacancies, Vacancy Clusters and Interparticle Spaces (with Diameters of Roughly 0.8 and 5 nm; Denoted as NP Spaces) in the CuO SA-NP Films, as Prepared by EPD from the CuO SA-NPs with Different Surface States (i.e., as-Washed, O_3_@RT, O_2_@300°C, O_3_@300°C)^a^

a The relative intensity describes the relative proportion of a specific lifetime component in the total number of measured annihilation events. The defect analysis was done for the measured PALS spectra, which were recorded at implantation energies up to 18 keV, corresponding to a maximum probing depth of a few micrometers.^87^ The CuO SA-NP films have a similar uniform thickness of 25–35 μm. A CuO SA-NP film prepared from as-washed NPs and post-annealed in air for 30 min at 200 °C was also measured. The estimated intensity uncertainty is within 5%.

The two short lifetime components (τ_1_ ≈ 204 ps and τ_2_ ≈ 401 ps; see Table S6.1) are by far the most dominant signal in the recorded PALS spectra: see Table 2. Positron lifetimes in the range of 180–190 ps for CuO NPs have been attributed to a mixed contribution from the annihilation of trapped positrons in the bulk and single vacancies.^83^ High-temperature annealing in the range to 1000–1175 °C induces pronounced CuO NP sintering and grain growth, resulting in a reduced lifetime in the range of 147–165 ps,^83,84^ which is within the expected range of 110–175 ps for simple single-crystalline bulk oxides, like CuO. For Cu_2_O films grown by atomic layer deposition (ALD), longer bulk and Cu-vacancy positron lifetimes were reported, as supported by DFT calculations.^85^ This suggest that, the by far most dominant short positron lifetimes τ_1_ and τ_2_ in our CuO EPD deposits, are likely a mixture of monovacancies and larger vacancy clusters, respectively. The minor contributions of the much longer lifetime components (τ_3_ and τ_4_) are tentatively attributed to interparticle spaces of roughly 1 and 5 nm size^86^ (having an intensity lower than ≤2%). In summary, PALS primarily resolves point defects for our CuO EPD film deposits; other methods are required to quantify micro/mesopores, which dominate the film porosity at much larger scales, e.g., as observed by SEM in Figure 7.

The PALS analysis in Table 2 indicates a distinct difference between the relative amounts of monovacancies and vacancy clusters in the annealed and nonannealed surface-treated CuO SA-NPs (see also Figure S6.2 for the variation of monovacancies only). The CuO SA-NP films formed from the as-washed and O_3_@RT-treated SA-NPs have a relatively higher amount of monovacancies (≈61 ± 1%) and a relatively lower amount of vacancy clusters (≈37 ± 1%) as compared to the films formed from the O_2_@300°C and O_3_@300°C SA-NPs (with near-equal monovacancy and vacancy cluster intensities of ≈50 ± 5%). These findings by PALS align with the XPS results, which evidence higher $\frac{\left[\mathrm{Cu}\left(I\right)\right]}{\left[\mathrm{Cu}\left(\mathrm{II}\right)\right]}$ valence-state fractions in the NP (sub)­surface for as-washed/ozonated SA-NPs as compared to annealed CuO SA-NPs.^10^ Minor residual fractions of near-surface Cu­(I) species in the nonannealed films identified by XPS^10^ thus correlate with a higher amount of isolated monovacancies by PALS. Air-annealing treatments should promote thermally assisted vacancy coalescence into larger vacancy clusters with concurrent oxidation of residual Cu­(I) to Cu­(II) species, thereby sharpening the SSC region, enhancing the surface potential and raising the ζ-potential (see Figure 5). This may even partially rationalize the consistently higher (discharge) current densities for EPD using the O_2_@300°C and O_3_@300°C nanopowders: see Figure 6.

To confirm such thermally assisted annealing of monovacancies, a CuO SA-NP film prepared from the as-washed NPs was postannealed in air at 200 °C and subsequently measured by XPS and PALS. XPS analysis indeed evidence the oxidation of Cu­(I) to Cu­(II) surface species by such postannealing: i.e., the Cu 2p_3/2_ main peak shifts to lower BEs, as accompanied by a decrease in the peak-to-satellite area ratio.^10^ The oxidation of Cu­(I) to Cu­(II) surface species during postannealing, as evidenced by XPS, thus indeed seems to correlate with a decrease in monovacancies (and concurrent increase in vacancy clusters) by PALS: see Table 2.

## Conclusion

Efficient, reproducible and up-scalable Electrophoretic Deposition (EPD) of oxide nanoparticles (NPs) from nonaqueous NP dispersions requires excellent colloidal stability and a sufficiently high NP surface charge at high NP loadings in solution. In this study, a surfactant-free approach is presented to optimize the colloidal stability and electrophoretic mobility of CuO NPs in nonaqueous media by tuning the NP surface space-charge (SSC) region in the dry state. To this end, various commercial and in-house synthesized CuO nanopowders (with unknown storage and atmospheric aging histories) are subjected to mild, organic-free surface treatments, including alternating washing steps in water and ethanol, as well as subsequent annealing in O_2_ or O_3_ at 300 °C.

Aged CuO nanopowders produce similarly low ζ-potentials in ethanol NP dispersions of about <| ± 20| mV, which is well below the threshold where EPD can still be performed. The formation of organic-oxygen species on the NP oxide surface during atmospheric aging, as well as by ozone oxidation at 300 °C, is accompanied by the creation of defective oxygen species in the NP subsurface region and an associated distortion of [CuO_6_] nearest-neighbor coordination spheres in CuO. Consequently, a redistribution of net surface charge from the outer surface toward the subsurface occurs, which results in a broadening of the SSC region and a correspondingly lower effective surface potential. The build-in electrostatic potential at the CuO NP surface will be screened by the electrical double layer in solution, dictating the ζ-potential. A broadening of the SSC region with an associated decrease of the built-in surface potential therefore also reduces the ζ-potential of the CuO NPs in solution.

The ζ-potential of the CuO NPs in ethanol can be very effectively increased by alternating washing steps in water and ethanol, as well as by subsequent annealing in O_2_ at 300 °C; its value may even exceed >| ± 100| mV in the absence of bulk impurities (e.g., Zn) in the CuO NP lattice. These mild, organic-free surface treatments restore the purity and crystallinity of the CuO NP surface, which sharpens the SSC, thereby increasing both the built-in surface potential and the measured ζ-potential in solution. As such, even at high NP loadings, excellent colloidal stability and high electrophoretic mobility are achieved, which enables the formation of coherent, uniform, porous CuO coatings with micrometer-scale thickness by EPD, which are entirely free of organic contaminants. Capacitative discharging of the deposited CuO NPs at the electrode surface result in a steady non-Faradaic current during the main deposition stage, as governed by the intrinsic surface defectiveness. In this regard, PALS analysis indicates a distinct difference between the relative amounts of monovacancies and vacancy clusters in the annealed and nonannealed surface-treated CuO SA-NPs. Annealing treatments promote thermally assisted vacancy coalescence into larger vacancy clusters (as accompanied by an oxidation of residual Cu­(I) to Cu­(II) valence states), thereby sharpening the SSC region, resulting in higher ζ-potentials and higher steady EPD discharge current densities. Nonpristine (i.e., contaminated or aged) CuO NP surfaces result in a much lower ζ-potential and no or highly incoherent EPD film formation. The uniformity, morphology and porosity of the EPD films can be tailored by the shape and size distribution of the original nanopowder, being largely independent of the NP surface pretreatment.

In summary, this study demonstrates a strong correlation between the surface defectiveness of dry CuO nanopowders, their effective surface potential, and their resulting ζ-potential in ethanol solution. Our experimental findings by XPS, ζ-potential measurements, PALS and EPD, all align with our main conclusion that even tiniest alterations of the surface defectiveness of the dry CuO NPs modify their electrokinetic behavior when dispersed in ethanol solvent and, consequently, their EPD performance. Admittedly, a full quantitative description of the dependence of ζ-potential on the surface state (i.e., surface defect structure and presence of organic-C adsorbates, giving rise to modified surface space-charge regions and effective surface potentials) is still out of reach. Accordingly, the proposed strategy is not claimed to be independent of all possible oxide nanopowder histories–especially not for those with bulk impurities–but should rather apply to multivalent oxide nanoparticles whose bulk composition is chemically pure and whose performance is mainly limited by surface aging and/or surface contaminants. Nevertheless, our findings may contribute to advance the development of efficient, scalable, and environmentally friendly EPD manufacturing routes for organic-free NP-based oxide coatings and membranes.

## Experimental Section

### CuO Nanopowders

Two commercial CuO nanopowders, purchased from US Research Nanomaterials, Inc. (catalogue number US3070) and Sigma-Aldrich (catalogue number 544868), were used, herein referred to as **US-NP** and **SA-NP**, respectively. Additionally, an additional CuO nanopowder was synthesized in-house using the sol–gel method, following the procedure outlined in ref 7, further denoted as **Empa-NP**.

The bulk CuO phase constitution of all three nanopowders was confirmed by X-ray diffraction (XRD) analysis using a Malvern PANalytical Empyrean with a Cu–Kα radiation source operating in a Bragg–Brentano geometry. Transmission electron microscopy (TEM; FEI Titan Themis 200 probe corrected from ThermoFisher) operated in bright-field mode at an accelerating voltage of 200 kV was applied to determine the morphology and NP size range of the nanopowders. To this end, the NPs were dispersed in ethanol and transferred onto a lacey carbon grid (300 mesh, TED PELLA, INC). NPs size ranges were measured using the ImageJ software (https://imagej.net; Madison, Wisconsin); 240 measurements were taken of individual particles and averaged. The specific surface areas (SSAs) of the nanopowders were determined by the Brunauer–Emmett–Teller (BET) method using a 5-point N_2_ adsorption BET isotherm, as measured with a Belsorp Mini X from Microtrac. Prior to the BET analysis, the powders were degassed for 2 h at 180 °C and 10 Pa.

The chemical states of the CuO NP surfaces after aging, as well as after different surface treatments, were studied by XPS using a PHI Quantes spectrometer (ULVAC-PHI) with a monochromatic Al–Kα X-ray source (*h*ν = 1486.6 eV, power 51 W, beam diameter 200 μm, detection angle of 45°). The energy scale of the hemispherical analyzer was calibrated according to ISO standard 15472 (2nd ed., 2010–05–01) by referencing the Au 4f_7/2_, Ag 3d_5/2_ and Cu 2p_3/2_ main peaks to their recommended binding energy (BE) positions of 83.96, 368.21, and 932.62 eV, respectively (as in situ measured for the sputter-cleaned, high-purity metal references). The nanopowders were pressed on a sticky carbon tape (as attached to a stainless-steel holder) in an under-pressurized Ar glovebox system (O_2_ ≤ 0.1 ppm, H_2_O ≤ 0.2 ppm) and transferred without intermediate air exposure for subsequent XPS analysis. The UHV chamber of the XPS instrument is directly coupled to the glovebox to safely accept the dry nanopowder samples through a load-lock system. The sticky carbon tape gives a characteristic signal from Si in the XPS analysis, which is not detected if the selected XPS analysis area is densely covered with the manually gently pressed nanopowder; this criterion was used for selecting the XPS analysis areas (having a diameter of 200 μm, see above). Survey scans of the selected areas were acquired with a pass energy of 224 eV and a step size of 0.8 eV. Next, high-resolution scans of the Cu 2p, C 1s, and O 1s regions were measured with a pass energy of 69 eV and a step size of 0.125 eV. Charge neutralization during XPS analysis was accomplished by dual-beam charge neutralization, employing low-energy electron and Ar ion beams (1-V bias, 20-μA current).

### CuO Nanopowder Treatments

The as-received CuO nanopowders (further denoted as **“as-received”**) were stored in their original vessel in an Ar glovebox (O_2_ ≤ 0.6 ppm, H_2_O ≤ 0.2 ppm) directly upon delivery (or after synthesis for the in-house nanopowder), thus preventing uncontrolled surface reactions with the laboratory environment during storage. A dedicated washing procedure (further denoted as **“as-washed”**; see ref 10) was applied to remove surface contaminants introduced during synthesis or aging, consisting of three repeated ultrasonic cleaning cycles with Milli-Q water­(with a conductivity of σ = 0.055 μScm^–1^ at 25 °C) followed by absolute ethanol (Sigma-Aldrich, ≥99.8%). One subsequent surface treatment (i.e., after washing, which was always performed as a first step before any other surface treatment) involved subsequent thermal annealing of the as-washed nanopowder in a tube furnace under synthetic air flow (0.3 L min^–1^, 20 vol % O_2_/N_2_) at 300 °C, further designated as **“O**_**2**_ **@300**°**C”** treatment. In addition, a similar annealing treatment of the as-washed nanopowder in a tube furnace at 300 °C was performed under continuous ozone flow, further denoted as **“O**_**3**_ **@300**°**C”** treatment. The ozone concentration was monitored both at the inlet and outlet of the tube furnace (using Ozone Analyzer BMT 965ST) to trace the thermal decomposition of ozone species. The initial inlet ozone concentration prior to heating was set to 70 g Nm^–3^ (approximately 0.06 g L^–1^). The annealing treatments were performed with a ramp-up rate of 10 °C min^–1^, a dwell time of 10 min at the target temperature, and subsequent water cooling of the tube flanges under continuous gas flow. A progressive drop in outlet ozone concentration was observed upon heating the tube furnace to the targeted temperature: i.e., from 70 g Nm^–3^ at room temperature (RT) to 5 g Nm^–3^ at 100 °C, to zero upon reaching the targeted temperature of 300 °C. The O_3_ annealing treatment at 300 °C differs from the room-temperature UV treatment in ozonated air (further referred to as **“O**_**3**_ **@RT”**, as detailed in ref 10), which mainly acts on the outer nanopowder bed surface.

### CuO Nanoparticle Dispersions

To prepare the CuO NP dispersions, a weighted amount of CuO nanopowder was dispersed in absolute ethanol (Sigma-Aldrich, ≥99.8%), which was subsequently ultrasonicated to disrupt weakly linked agglomerates (using a Sonopuls HD 4200 from Bandelin). All CuO dispersions were prepared with a default CuO NP concentration of 5 g L^–1^ (0.6 wt %). The optimized settings for the ultrasonic treatment were as follows: 10 min in ultrasonic bath and 10 min with the sonotrode (US-Homogenizator, Sonopuls GM4200) operated in pulsing mode (2 s burst, 2 s break). The sonication process was carried out in an ice bath to avoid possible reduction of CuO by ethanol.^10^ Unless otherwise stated, all dispersions were prepared via this procedure.

The ζ-potentials of dispersed CuO NPs in ethanol were measured at a particle concentration of 0.6.% by the multifrequency (300 kHz-3 mHz) electroacoustic method using a Zeta Probe instrument (Colloidal Dynamics Inc., North Attleboro). The instrument measures the electrokinetic sonic amplitude (ESA), converts it into a frequency-dependent dynamic mobility spectrum, and calculates the ζ-potential by fitting this spectrum using the O’Brien electroacoustic model for concentrated colloidal dispersions.^88^ The calculation included the relevant material and solvent parameters, including the dielectric constants of CuO and ethanol, as well as the density and viscosity of ethanol.

For each CuO NP state, three independent measurements were performed and averaged. Each individual measurement consisted of ten data points collected over 10 min, which were averaged prior to analysis. To estimate the uncertainty associated with the concentration-dependent ESA-to-ζ conversion, the measured dynamic mobility spectrum obtained at 0.6% was additionally recalculated using particle concentrations of 0.5 and 0.7%. The resulting variation was incorporated into the reported error bars. Consequently, the larger absolute uncertainties observed for dispersions exhibiting high absolute ζ-potentials reflect the increased sensitivity of the electroacoustic model to particle concentration at high dynamic mobility, rather than measurement accuracy and reproducibility.

### Electrophoretic Deposition (EPD)

Electrophoretic deposition (EPD) from the CuO NP dispersions was conducted using a PLH250 power supply from Aim-TTi. Current flowing through the EPD cell was measured via a shunt resistor. The voltage drop across the shunt resistor was measured using a Keithley DMM6500 multimeter. A platinised Nb-mesh (METAKEM) and a FTO-glass slide (Sigma-Aldrich, 735140–5EA) with sizes of 10 × 50 mm^2^ were used as a counter electrode (EPD supporting electrode) and working electrode (electrode, where NP deposition took place), respectively. Electrodes were placed parallel at a 4 mm distance, while immersing an electrode area of 10 × 25 mm^2^ into the dispersion (Figure S1 in Supporting Information). The EPD step was carried out potentiostatically according to the deposition profile in Table 3.

**Table 3:** EPD Deposition Profile Adopted in This Work^a^

	Ramp up	Dwell *t*_1_ (s)	Ramp down	Dwell *t*_2_ (s)
Profile	to 25 V in 30 s	120	to 1 V in 1 s	60

a The number of EPD cycles was adjusted to the desired film thickness: 4 cycles resulted in ≈30 μm thick films; 2 cycles resulted in ≈15 μm thick films.

The morphologies of the as-received nanopowders and the deposited nanoporous CuO films were investigated using a ZEISS GeminiSEM 460 scanning electron microscope (SEM) equipped with an InLens SE detector for high-resolution imaging. The weight of the electrodes was noted prior to EPD to derive the deposited mass and film density (by estimating the film thickness). The film thickness was determined from SEM cross-sectional images using the vendor’s software. The cross sections were prepared by breaking the coated electrode along a precut line, followed by sputtering 5 nm of Au to ensure conductivity for HR-SEM imaging.

### X-ray Photoelectron Spectroscopy (XPS)

The chemical state of the CuO NP surfaces after aging, as well as after different surface treatments was studied by XPS analysis using a PHI Quantes spectrometer (ULVAC-PHI) with a monochromatic Al–Kα X-ray source (*h*ν = 1486.6 eV, power 51 W, beam diameter 200 μm, detection angle of 45°). The energy scale of the hemispherical analyzer was calibrated according to ISO standard 15472 (second edition, 2010–05–01) by referencing the Au 4f_7/2_, Ag 3d_5/2_ and Cu 2p_3/2_ main peaks to the recommended binding energy (BE) positions of 83.96, 368.21 and 932.62 eV, respectively (as in situ measured for the sputter-cleaned, high-purity metal references). The nanopowders were pressed on a sticky carbon tape (as attached to a stainless-steel holder) in an under-pressurized Ar glovebox system (O_2_ ≤ 0.1 ppm, H_2_O ≤ 0.2 ppm) and transferred without intermediate air exposure for subsequent XPS analysis. The sticky carbon tape gives a characteristic signal from Si in the XPS analysis, which is not detected if the selected XPS analysis area is densely covered with the manually gently pressed nanopowder; this criterion was used for selecting the XPS analysis areas (having a diameter of 200 μm, see above). Survey scans of the selected areas were acquired with a pass energy of 224 eV and a step size of 0.8 eV. Next, high-resolution scans of the Cu 2p, C 1s, and O 1s regions were measured with a pass energy of 69 eV and a step size of 0.125 eV. Charge neutralization during XPS analysis was accomplished by dual-beam charge neutralization, employing low-energy electron and Ar ion beams (1-V bias, 20 μA current).

### Positron Annihilation Lifetime Spectroscopy (PALS)

The defectiveness of deposited CuO films was investigated by variable-energy positron annihilation lifetime spectroscopy (VEPALS). Measurements were performed at the Monoenergetic Positron Source (MePS) beamline, a end station at the ELBE radiation source (Electron Linac for beams with high Brilliance and low Emittance) at HZDR, Germany.^89^ A CeBr_3_ scintillation detector coupled to a Hamamatsu R13089–100 photomultiplier tube (PMT) was used for γ photon detection. Signal acquisition was carried out using a Teledyne SP Devices ADQ14-DC-2X-MTCA digitizer, with a vertical resolution of 14 bits and a sampling rate of 2 GS/s.^90^ The overall time resolution of the measurement system was, ≈0.250 ns and all spectra contained at least 1 × 7 counts. A typical lifetime spectrum, *N­(t)* (i.e., the absolute value of the time derivative of the positron decay spectrum) is described by *N*(*t*) = *R*(*t*)*∑_*i* = 1_^*k*+1^[*I*_*i*_/τ_*i*_·e^–*t*/τ_*i*_^] + “Background”, where *k* is the number of different defect types contributing to positron trapping, which are related to *k* + 1 components in the spectra with individual lifetimes, τ_*i*_, and intensities, *I*_*i*_ (∑*I*_*i*_ = 1).^79^ The instrumental resolution function, *R­(t)*, is a sum of two Gaussian functions with distinct intensities and relative shifts both depending on the positron implantation energy, *E*_p_. It was determined by measurement and analysis of a Yttria-stabilized zirconia (YSZ) reference sample with a single well-known lifetime component. The background was negligible, hence fixed to zero.

The measured spectra were deconvoluted using a nonlinear least-squares fitting method, minimized by the Levenberg–Marquardt algorithm, using the PALSfit software package,^82^ while adopting two major (i.e., dominant) and two minor lifetime components, representing localized annihilation at four different defect types (sizes; τ_1_, τ_2_, ···). The corresponding relative defect intensities scale with the respective defect concentrations. The positron lifetime generally increases with defect (open volume) and pore (free volume) size. The positron lifetime and average positron lifetime (defined as τ_av_ = ∑_*i*_ τ_*i*_·*I*_*i*_) and its corresponding intensity were probed as a function of positron implantation energy *E*_p_.

## Supplementary Material



## Notes

(S1) Experimental setups; (S2) XPS analysis of as-received, aged, and treated CuO nanopowders; (S3) XPS analysis of CuO films grown by electrophoretic deposition; (S4) CV scan of CuO SA-NP dispersion; (S5) CuO film microstructures after EPD; (S6) PALS analysis of the EPD CuO films; (S7) Control experiment for background in pure ethanol; (S8) Electrophoretic deposition from CuO NP dispersion prepared with freshly opened ethanol; (S9) Estimation of particle–particle interactions in highly loaded CuO NP ethanol dispersions (PDF)
