# Supplementary material for: Surfactantless Electrophoretic Deposition of Nonaqueous CuO Nanoparticle Dispersions by Tailoring Their Surface Defectiveness
Source: Langmuir. 2026 Jul 23;42(30):21965–80. doi: 10.1021/acs.langmuir.6c01947 (PMC13450482; doi:10.1021/acs.langmuir.6c01947)
Supplement: Supplementary file 1 [file la6c01947_si_001.pdf]

Supporting Information

Surfactantless electrophoretic deposition of non-aqueous CuO nanoparticle dispersions by tailoring their surface defectiveness

Anastasia Batenkova[1,2], Jan P. Kollender[1], Eric Hirschmann[3], Maciej O. Liedke[3], Maik Butterling[3], Daniele Casari[4], Patrik Schmutz[1], and Lars P.H. Jeurgens*[1]

[1]Laboratory for Joining Technologies and Corrosion, Empa - Swiss Federal Laboratories for Materials Science and Technology, Dübendorf, 8600, Switzerland
[2]Department of Chemistry and Applied Biosciences, ETHZ - Federal Institute of Technology Zurich, Zurich, 8093, Switzerland
[3]Institute of Radiation Physics, Helmholtz-Zentrum Dresden – Rossendorf (HZDR), Dresden, 01328, Germany
[4]Laboratory for Mechanics of Materials and Nanostructures, Empa - Swiss Federal Laboratories for Materials Science and Technology, Thun, 3602, Switzerland

*Email: lars.jeurgens@empa.ch

Contents

(**S1**) Experimental setups
(**S2**) XPS analysis of as-received, aged and treated CuO nanopowders
(**S3**) XPS analysis of CuO films grown by electrophoretic deposition
(**S4**) CV scan of CuO SA-NP dispersion
(**S5**) CuO Film Microstructures after EPD
(**S6**) PALS Analysis of the EPD CuO Films
(**S7**) Control experiment for background in pure ethanol
(**S8**) Electrophoretic deposition from CuO NP dispersion prepared with freshly opened ethanol
(**S9**) Estimation of particle-particle interactions in highly loaded CuO NP ethanol dispersions.

S1 Experimental Setups

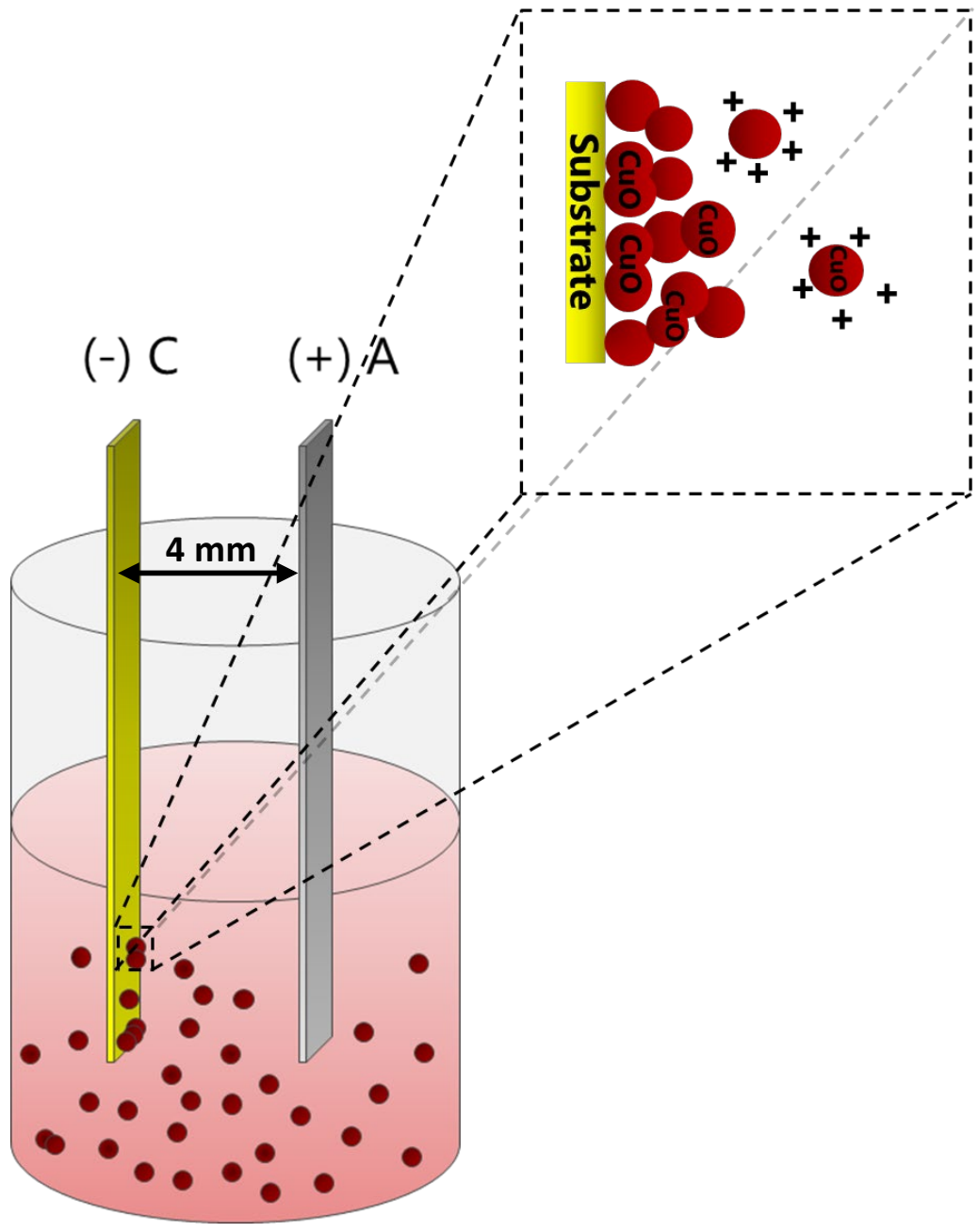

Figure S1.1 Sketch of the electrochemical cell used for electrophoretic deposition from the CuO NP dispersions on FTO-glass slides. C = cathode; A = Anode (platinized Nb-mesh).

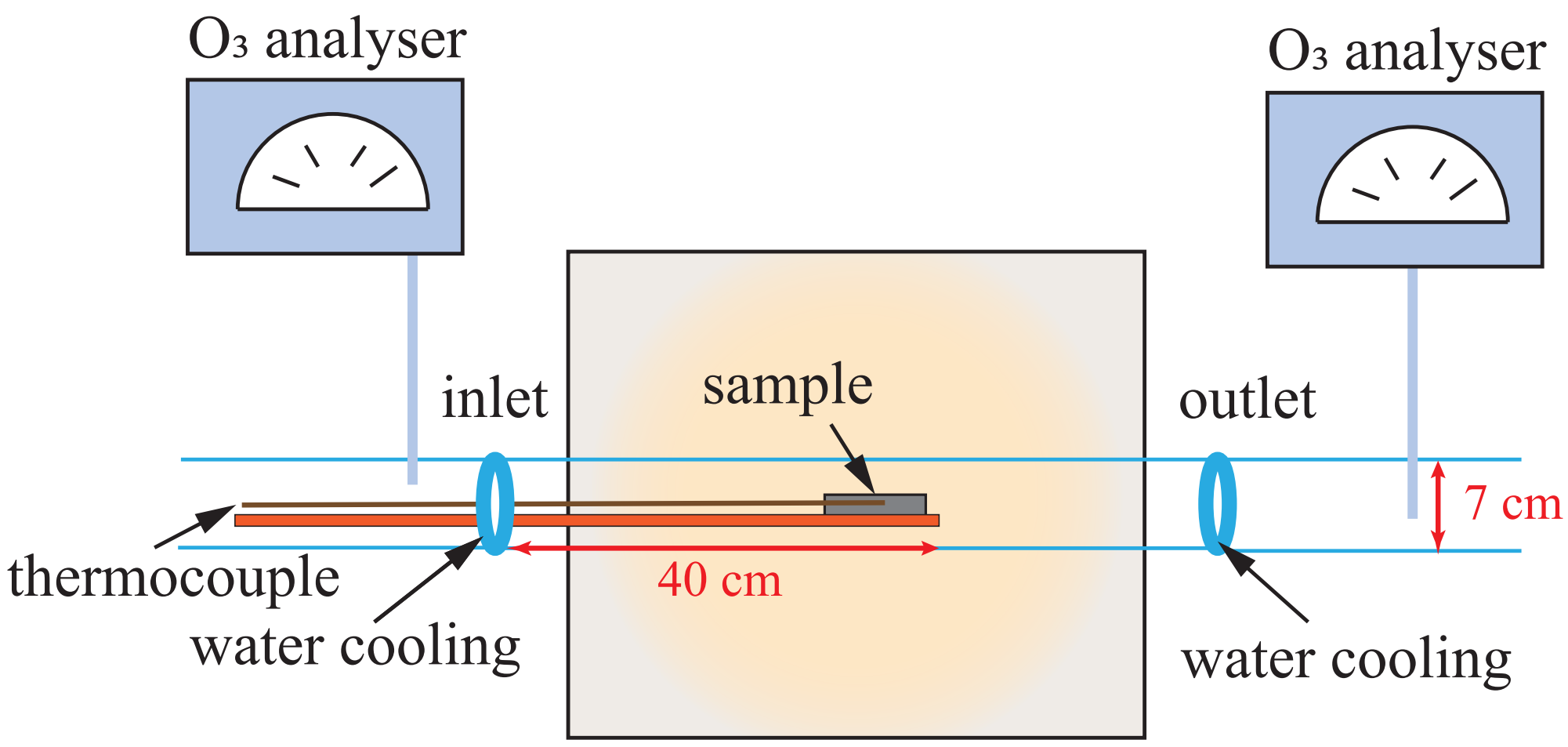

Figure S1.2 Schematic of the custom built tube furnace for ozone oxidation.

S2 XPS analysis of as-received, aged and treated CuO nanopowders

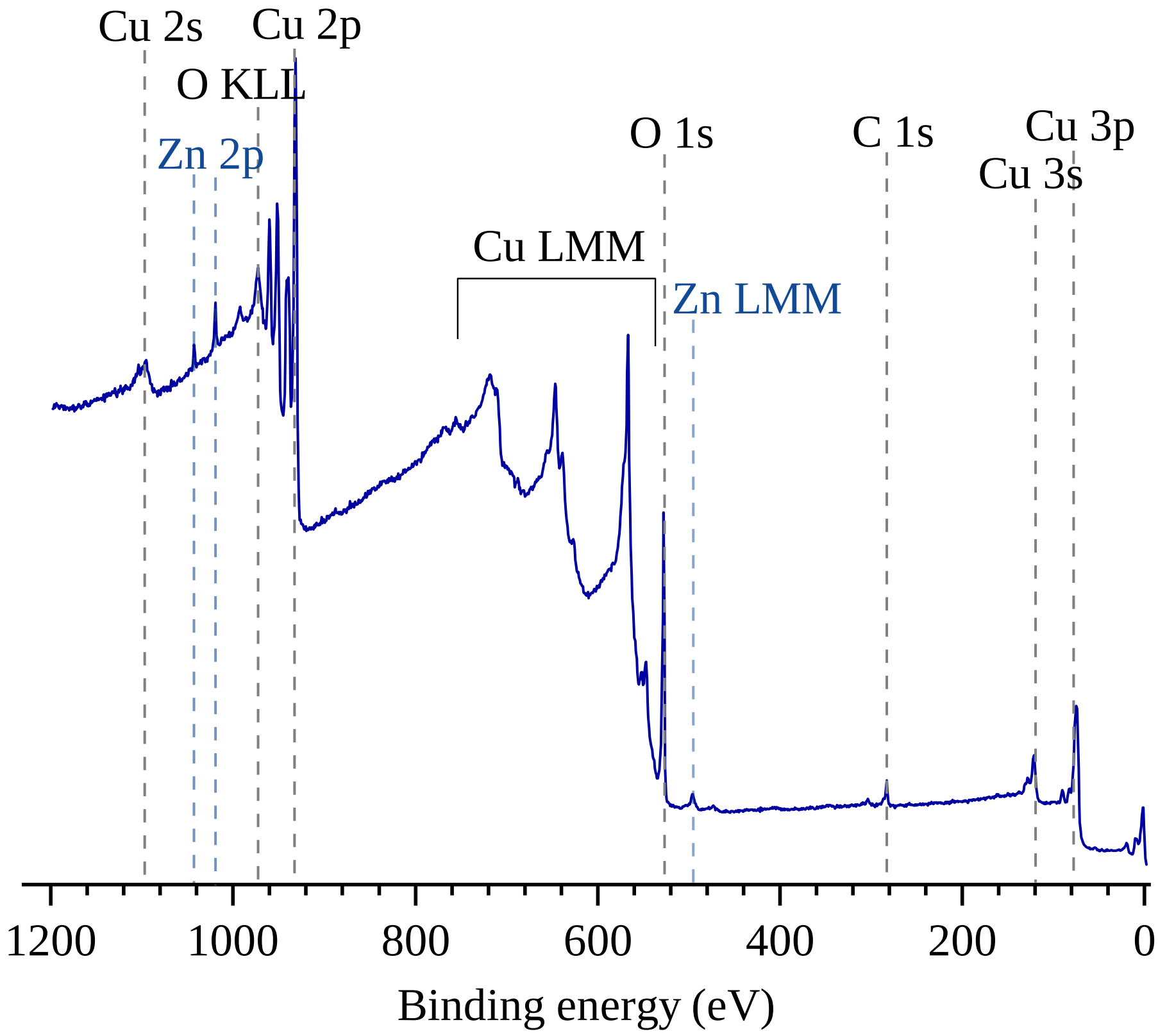

Figure S2.1 XPS survey of the as-washed CuO US-NP nanopowders, showing persistent Zn impurities, while Na impurities are effectively removed after washing.

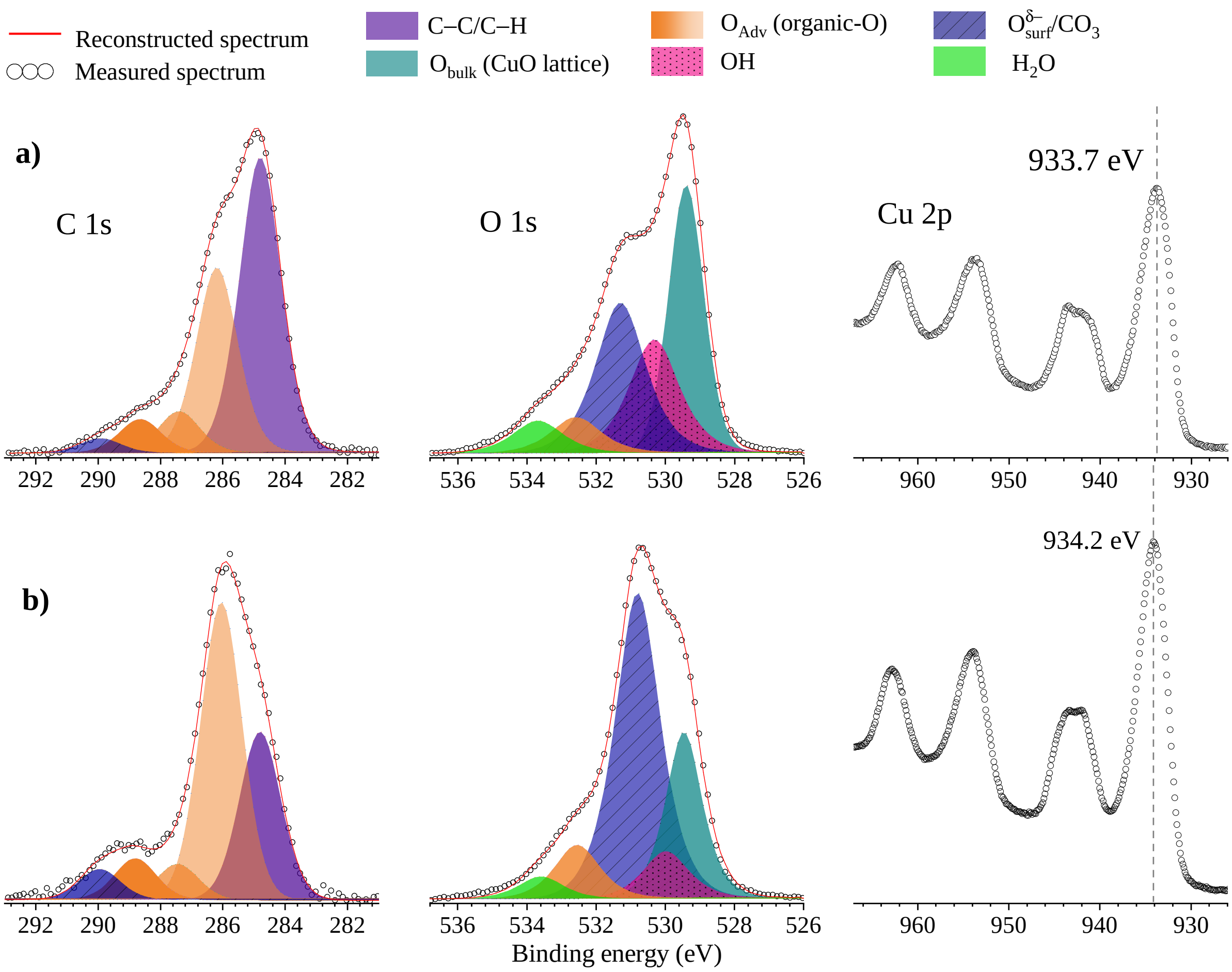

Figure S2.2 Measured and reconstructed XPS spectra of the C 1*s* region (*left column*), the O 1*s* region (*middle column*), and the Cu $2p_{3/2}$ region (*right column*) as recorded from the in-house synthesized Empa-NP: (**a**) for the as-synthesized state and (**b**) after annealing at 300 °C in O_3-flow (O_3@300 °C). The different chemical species, as resolved by constrained peak fitting of the C 1*s* and O 1*s* regions, are represented by specific colors with the different types of organic-O species being combined in an orange color scale. The imposed constraints for the chemical shifts and FWHMs of the fitted synthetic main peaks are reported in [1]. The C 1*s* and O 1*s* spectra shown were normalized by the integrated intensity of the Shirley background corrected spectral envelope to correct for variations in absolute photoelectron intensity, depending on the selected position and area for XPS analysis. The Cu $2p_{3/2}$ spectra is not normalized, reflecting the peak position and FWHM of the main peak and the shape of the respective satellite.

S3 XPS analysis of CuO films grown by electrophoretic deposition

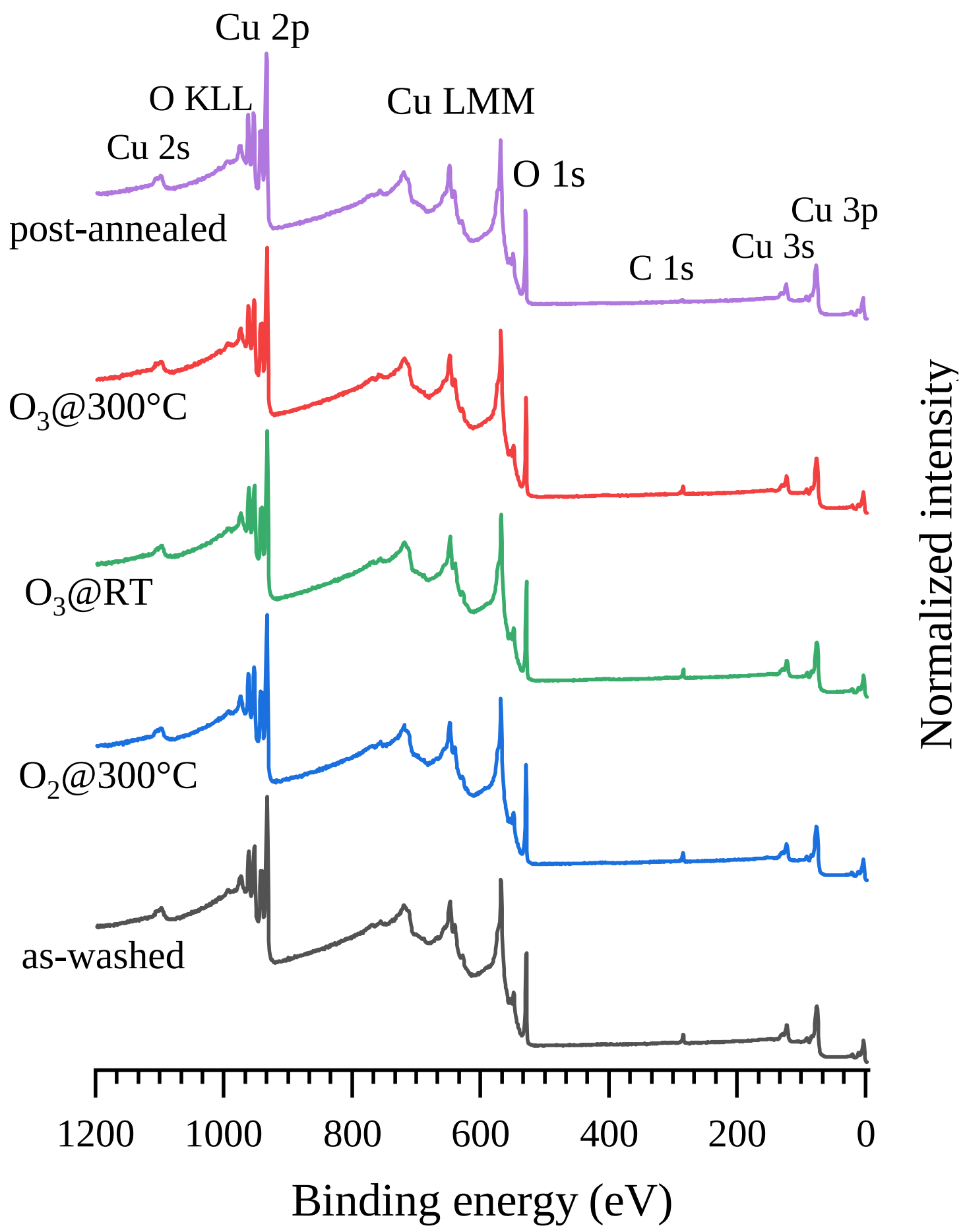

Figure S3.1 XPS survey spectra of deposited CuO SA-NP films for different surface treatment of the SA-NPs, i.e. as-washed, O_3@300 °C, O_3@RT, O_2@300 °C. In addition, an XPS survey of the CuO films prepared using EPD of the "as-washed" SA-NPs after annealing for 30 min at @200 °C is shown. Only signals from Cu, O and C are detected. Note that the adventitious carbon signal is relatively low.

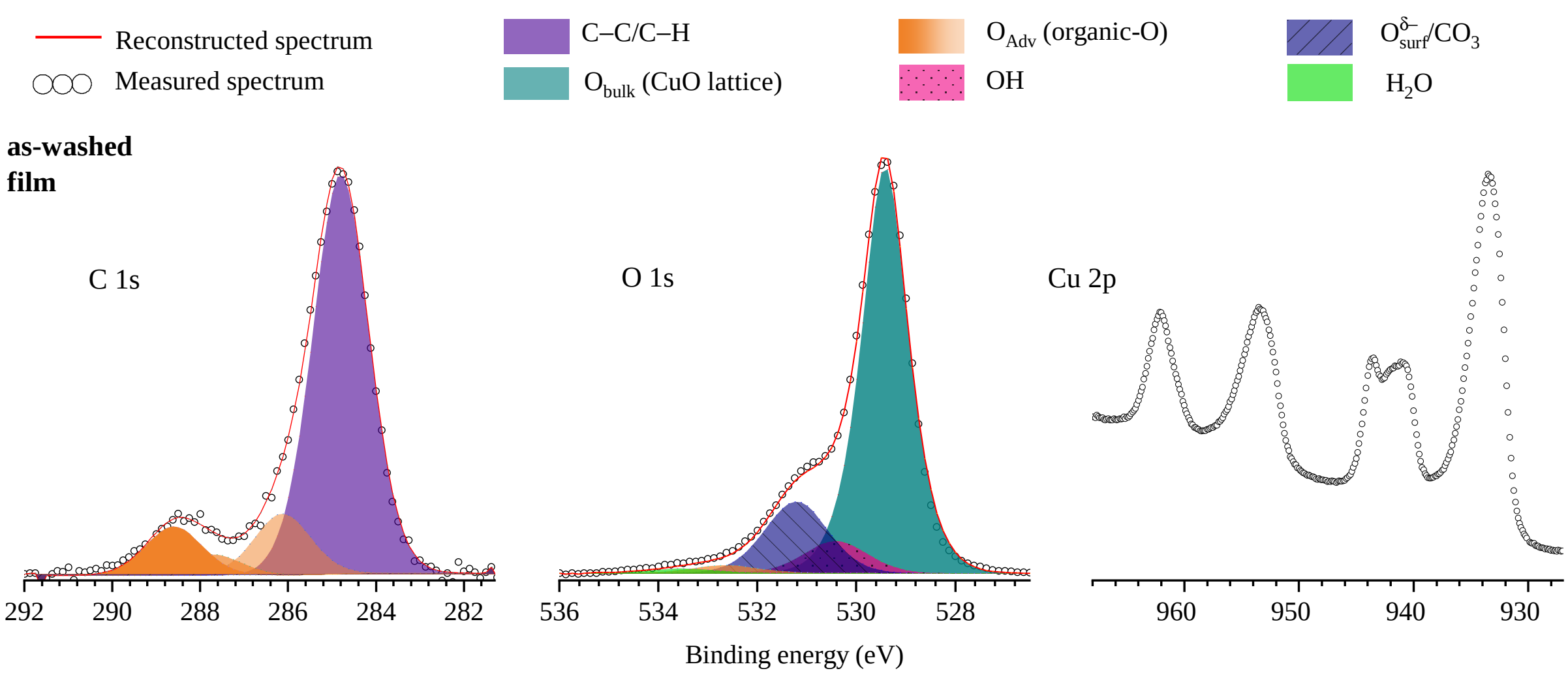

Figure S3.2 XPS HR spectra of deposited CuO SA-NP film for "as-washed" sample.

S4 CV scan of CuO SA-NP dispersion

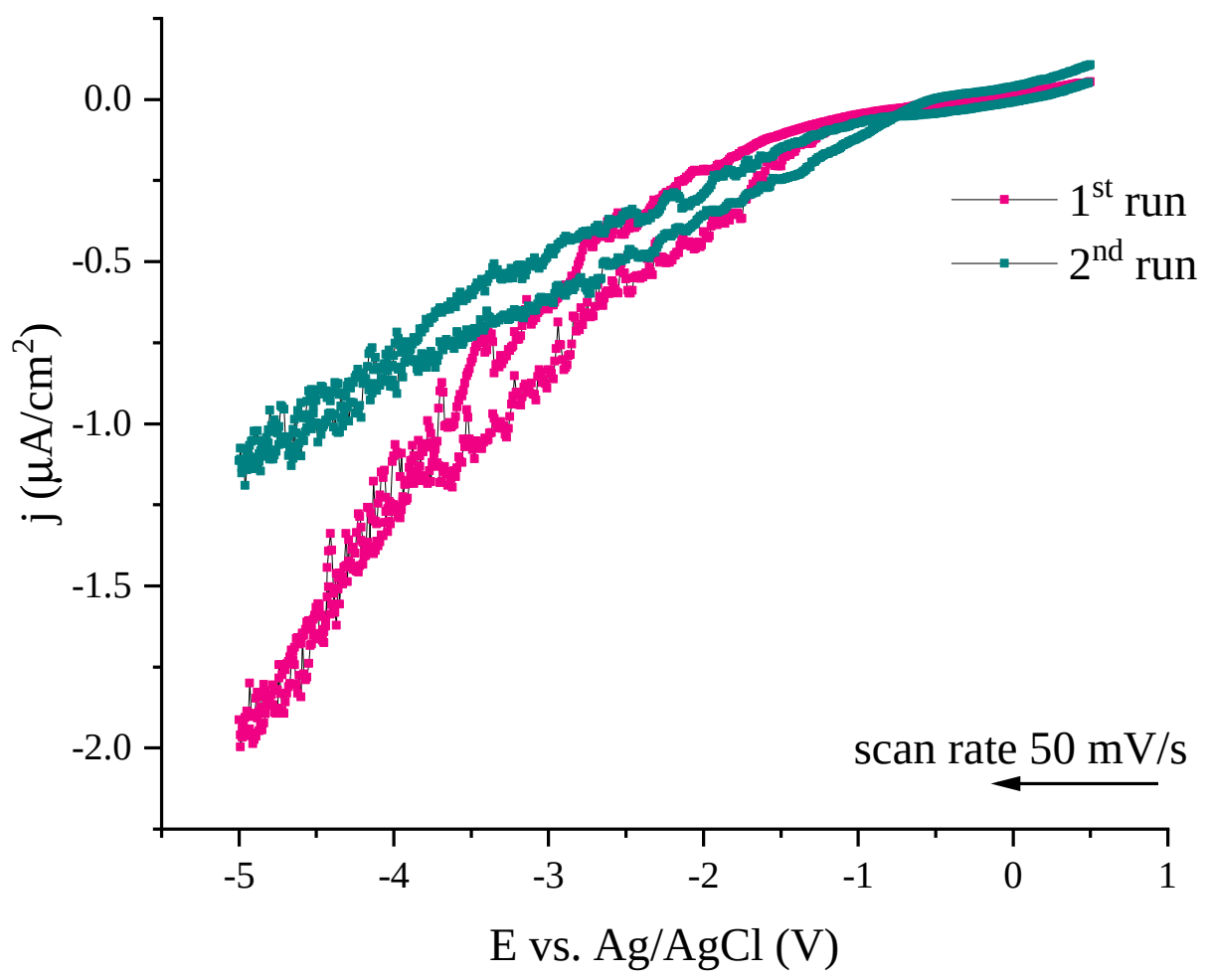

Figure S4.1 CV scan of CuO SA-NP dispersion demonstrates linear dependency of EPD current on the applied polarization, evidencing that CuO is not reduced in the applied potential range.

S5 CuO Film Microstructures after EPD

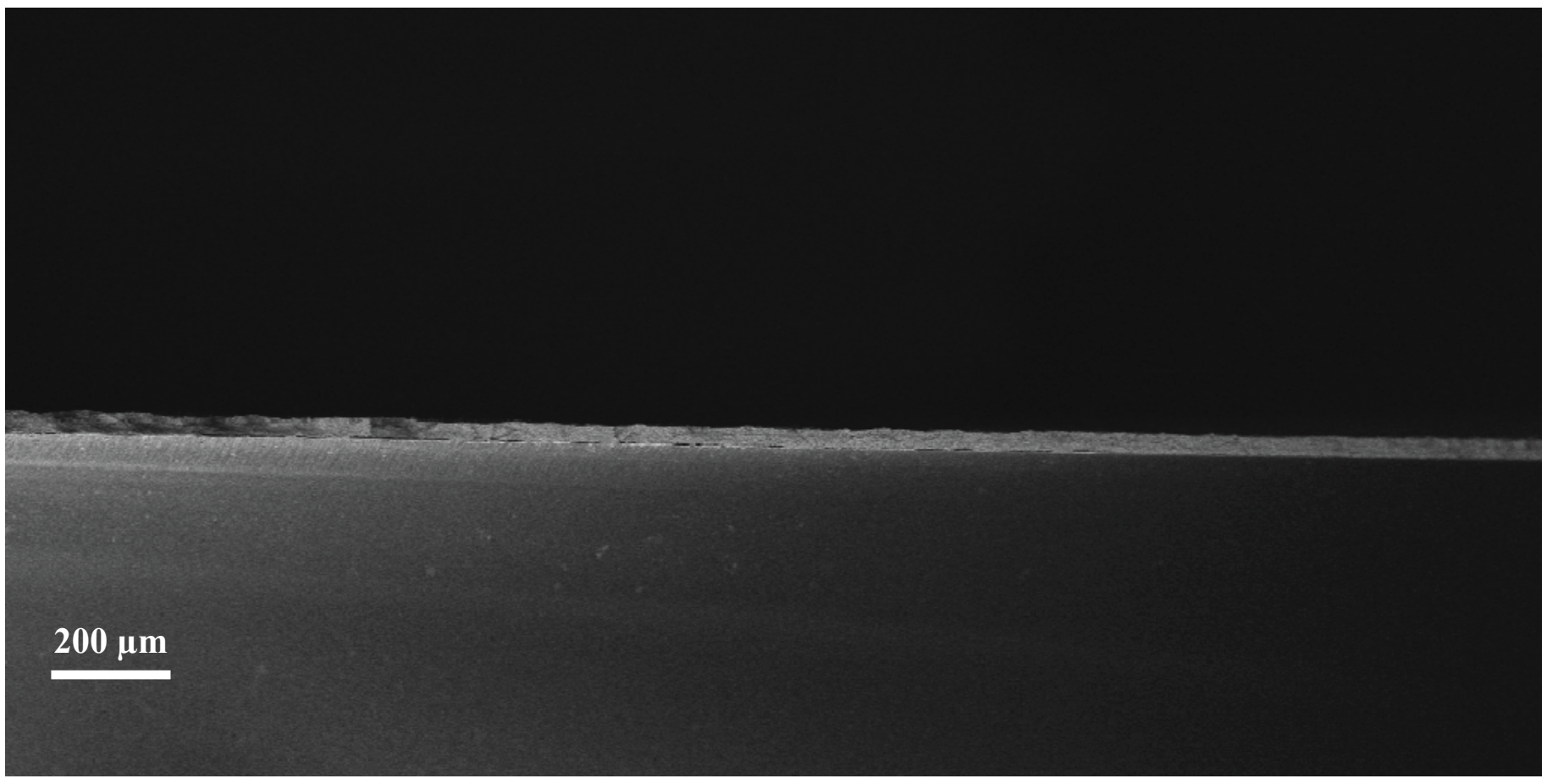

Figure S5.1 Cross-sectional SEM micrograph (at relatively low magnification) of a CuO film obtained by EPD (deposition profile; see Table 3) using "as-washed" SA-NPs. It demonstrates uniform substrate coverage with a uniform film thickness of ≈30 µm.

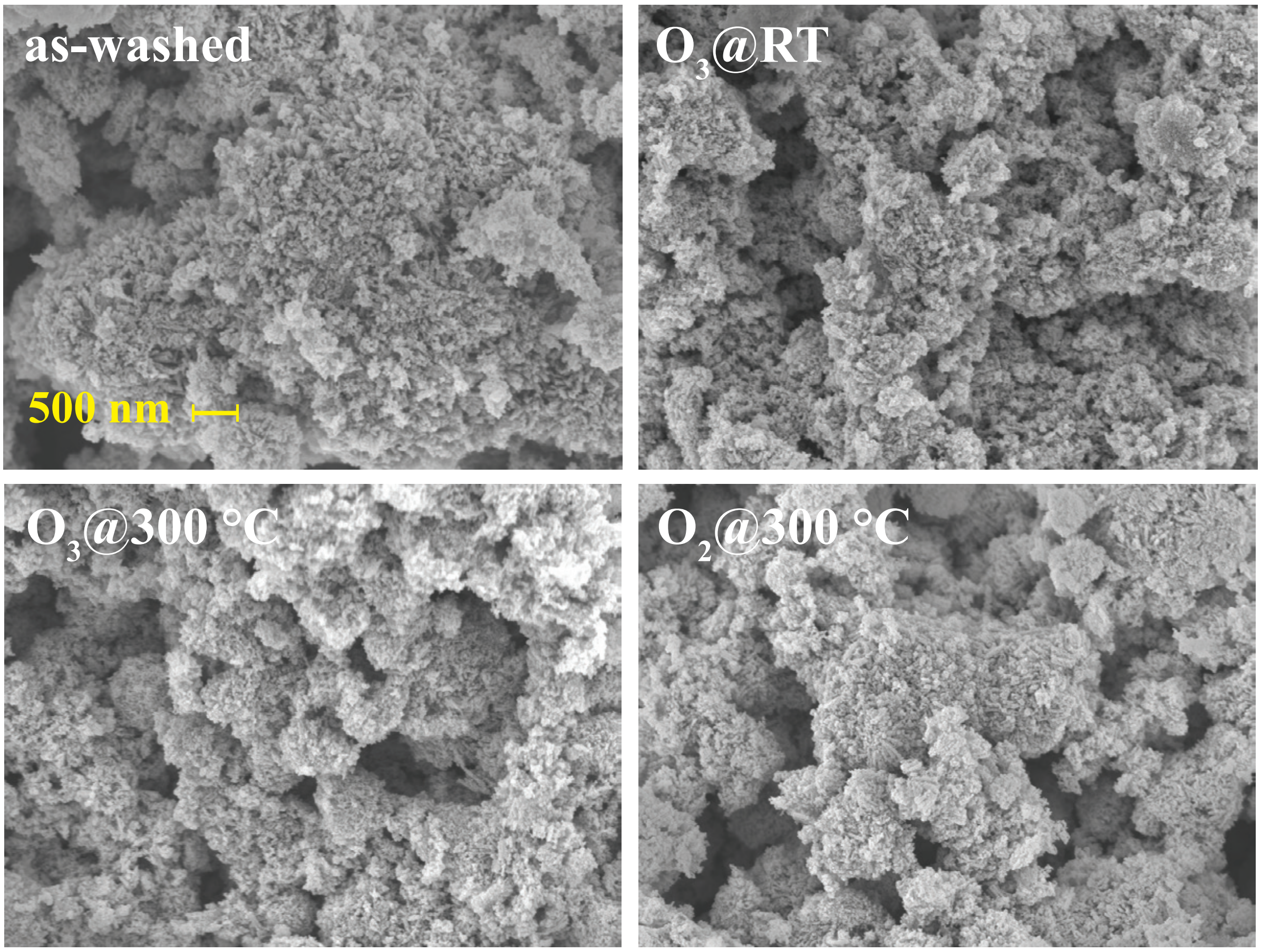

Figure S5.2 Cross-sectional SEM micrographs of CuO nanoporous films as prepared by EPD from CuO US-NPs dispersed in ethanol (see Table 3) for different surface-treatment of the as-washed CuO US-NPs.

S6 PALS Analysis of the EPD CuO Films

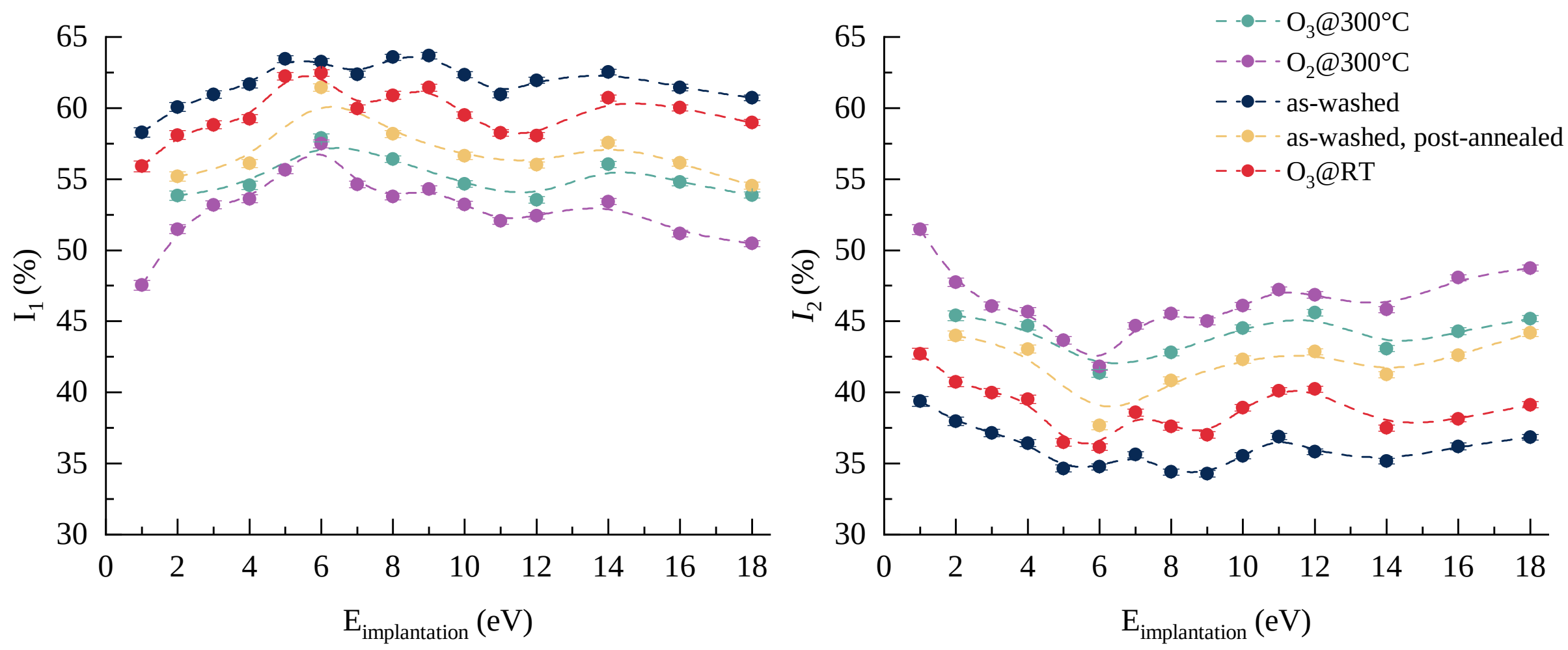

Figure S6.1 Relative intensities I_1 and I_2 correspond to positron lifetime components, τ_1 and τ_2, as obtained from the curve fitting of the measured PALS for implantation energies in the range of 2 up to 18 keV.

Table S6.1 Summary of defect distributions across samples (thickness 25 – 35 µm for a 14 min deposition time) as resolved by curve-fitting of the measured PALS spectra recorded from the different CuO EPD films [3].

**Life-Time Component**	τ_1 **[ns]**	τ_2 **[ns]**	τ_3 **[ns]**	τ_4 **[ns]**
Defect Type	monovacancies	vacancy clusters	interparticle spaces (0.8 – 1 nm)	interparticle spaces (4.6 – 5.2 nm)
	0.204	0.401	3.2 – 4.4	62 – 68

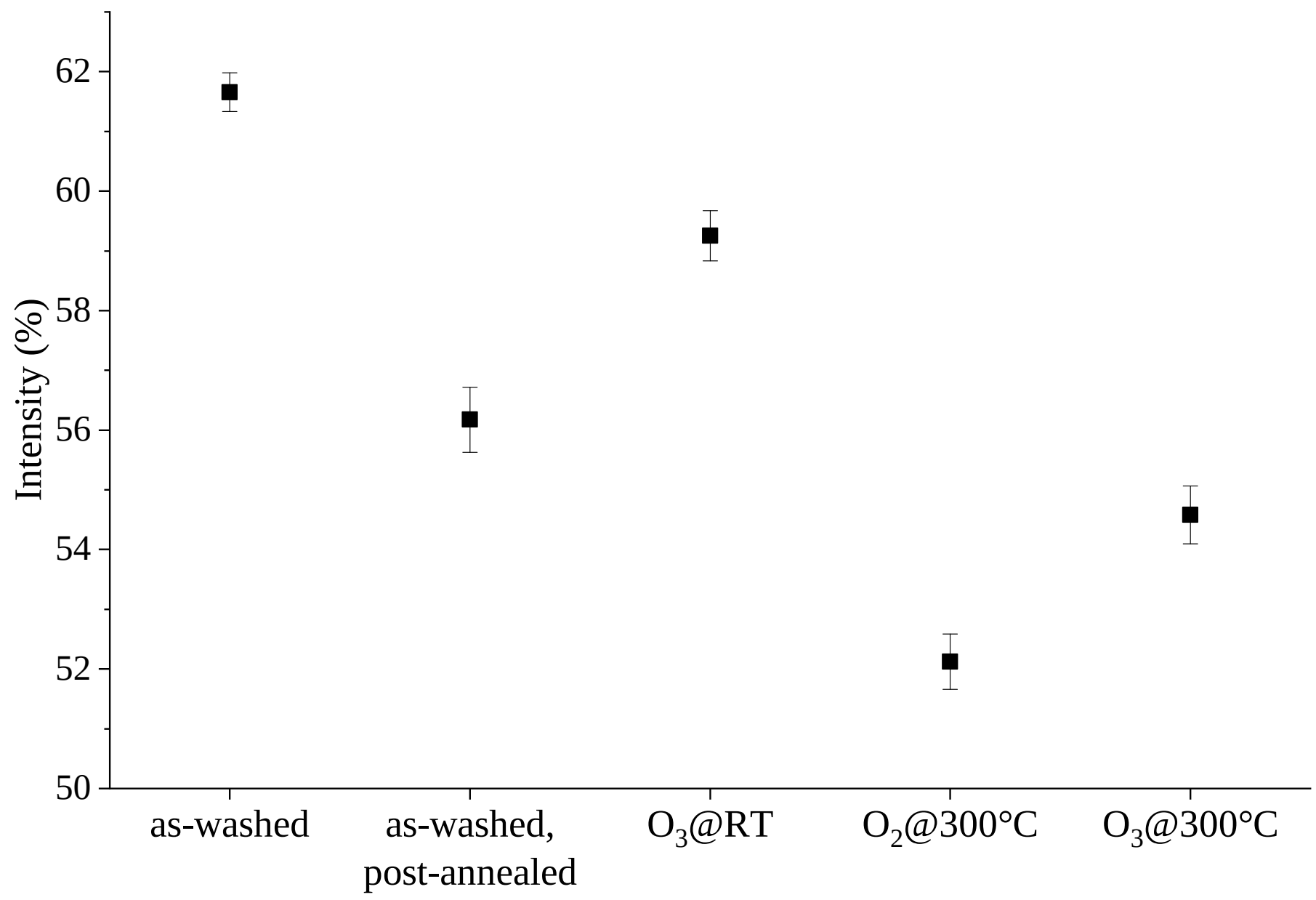

Figure S6.2 Relative intensity of the main positron lifetime component, τ_1, as obtained from the curve fitting of the measured PALS for implantation energies in the range of 10 up to 18 keV (corresponding to a maximum probing depth of a few micrometers [2]). The relative intensity describes the relative proportion of a specific lifetime component in the total number of measured annihilation events. It reflects the capture probability of positrons in a specific defect type. A higher intensity of a defect-specific signal correlates directly with a higher concentration of these defects in the material.

S7 Control experiment for background in pure ethanol

A control experiment was performed in pure ethanol under conditions identical to those used for EPD from the CuO nanoparticle dispersions in order to establish the background current density. The current density–time curves (bottom row) obtained in pure ethanol using the same FTO/Nb electrode configuration and applied voltage (top row) enable to distinguish between nanoparticle transport/deposition contributions and background electrochemical processes.

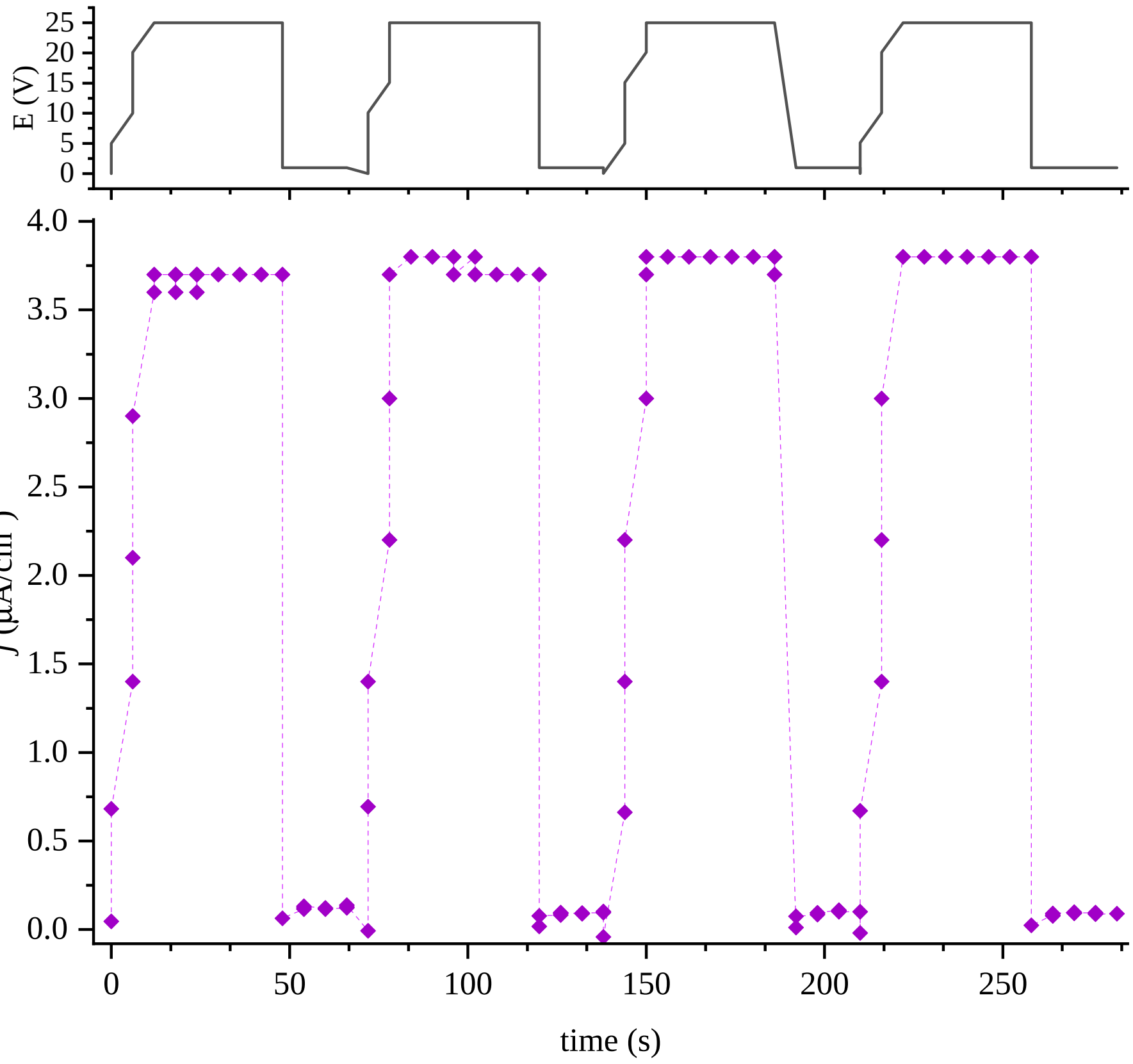

Figure S7.1 Current density–time curves recorded in pure ethanol (bottom row) with the same FTO/Nb electrode configuration and applied voltage (top row) as used for EPD from the CuO nanoparticle dispersions.

S8 Electrophoretic deposition from CuO NP dispersion prepared with freshly opened ethanol

The current density-time curves (bottom row) and applied voltage profiles (top row) are shown for electrophoretic deposition performed from a CuO NP dispersion prepared with freshly opened ethanol and treated CuO NPs. The current density is abnormally high, reaching approximately 40 µA. Typically, no deposit is obtained when the current density exceeds approximately 20 µA. Since the same ethanol was used to successfully deposit the films discussed in the manuscript, ethanol impurities or minor amounts of adsorbed water can be excluded as the primary factor controlling the success of EPD.

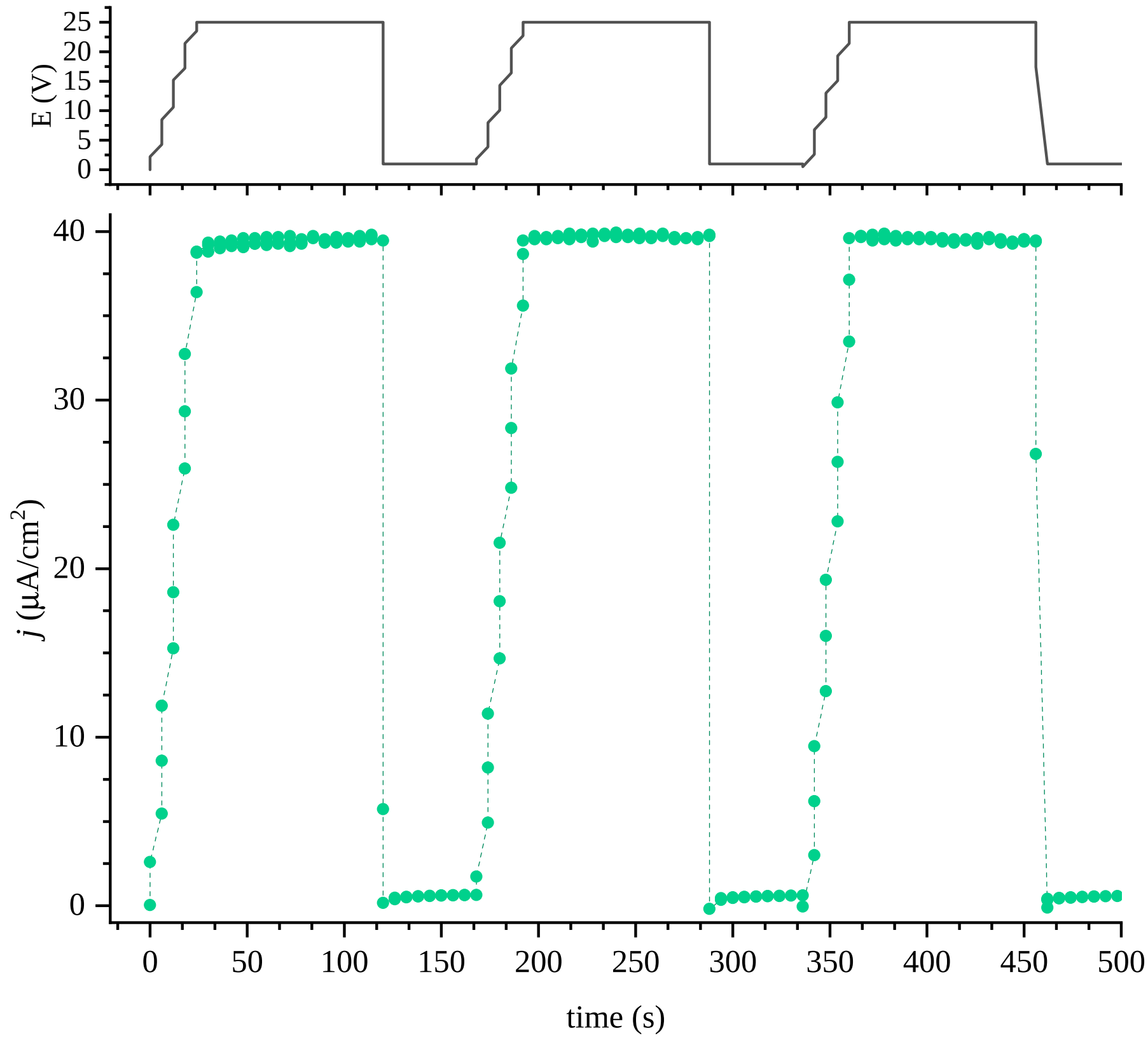

Figure S8.1 Current density-time curves (bottom row) and applied voltage profiles (top row) recorded during electrophoretic deposition from a treated CuO NP dispersion prepared with freshly opened ethanol.

S9 Estimation of particle-particle interactions in highly loaded CuO NP ethanol dispersions.

At very high oxide NP concentrations in ethanol as used for EPD (i.e. 5 g/L), electrical double layers and hydrodynamic fields (i.e. particle-particle interactions transmitted through the liquid flow field) interact, and many-body interactions between the NPs, need to be accounted for. As a result, the measured electrophoretic mobility becomes concentration-dependent: i.e. the system can no longer be described as isolated particles in ethanol solution. The contour plot in Fig. **S9.1** provides a dimensionless estimate of double-layer overlap, h/λ_D, in dependence of the CuO NP diameter and the CuO concentration, C_{CuO}, for CuO NPs dispersed in absolute ethanol at RT. The average distance between neighbouring nanoparticles in solution, h, can be estimated from the CuO mass concentration (in the range from 10^{-2} to 10^3 g/L) and the average NP diameter, assuming a CuO density of 6.31 g/cm^3. The Debye screening length, λ_D, depends on the relative permittivity ($\varepsilon_r \approx 24.3$) and the effective ionic strength ($I_{eff} \approx 10^{-8}$ to 10^{-5} mol/L) of absolute ethanol according to:

$$\lambda_D = \sqrt{\frac{\varepsilon_r \varepsilon_0 k_B T}{2 N_A e^2 \left(I_{eff} \cdot 10^3\right)}}, \tag{S1}$$

where ε_r is the relative permittivity of ethanol, ε_0 is the vacuum permittivity, k_B is the Boltzmann constant, $T = 298.15$ K is the temperature, N_A is Avogadro's number, e is the elementary charge, and I_{eff} is the effective ionic strength expressed in mol/L. The factor 10^3 converts I_{eff} from mol/L to mol/m^3.

The colour scale in Fig. **S9.1** shows the ratio h/λ_D with each panel corresponding to a different effective ionic strength of ethanol in the range of 10^{-8} to 10^{-5} mol/L. Low values of h/λ_D indicate that the electrostatic double layers of neighbouring particles overlap strongly, whereas high values indicate that the particles are effectively electrostatically isolated. The red shaded band indicates the transition regime, $1 < h/\lambda_D < 3$, for which the Debye length is comparable to the interparticle spacing. The red marker indicates the experimental condition for spherical CuO NPs with an average diameter of 50 nm dispersed in ethanol at 5 g/L (with $\varepsilon_r \approx 24.3$) at $T = 298.15$ K.

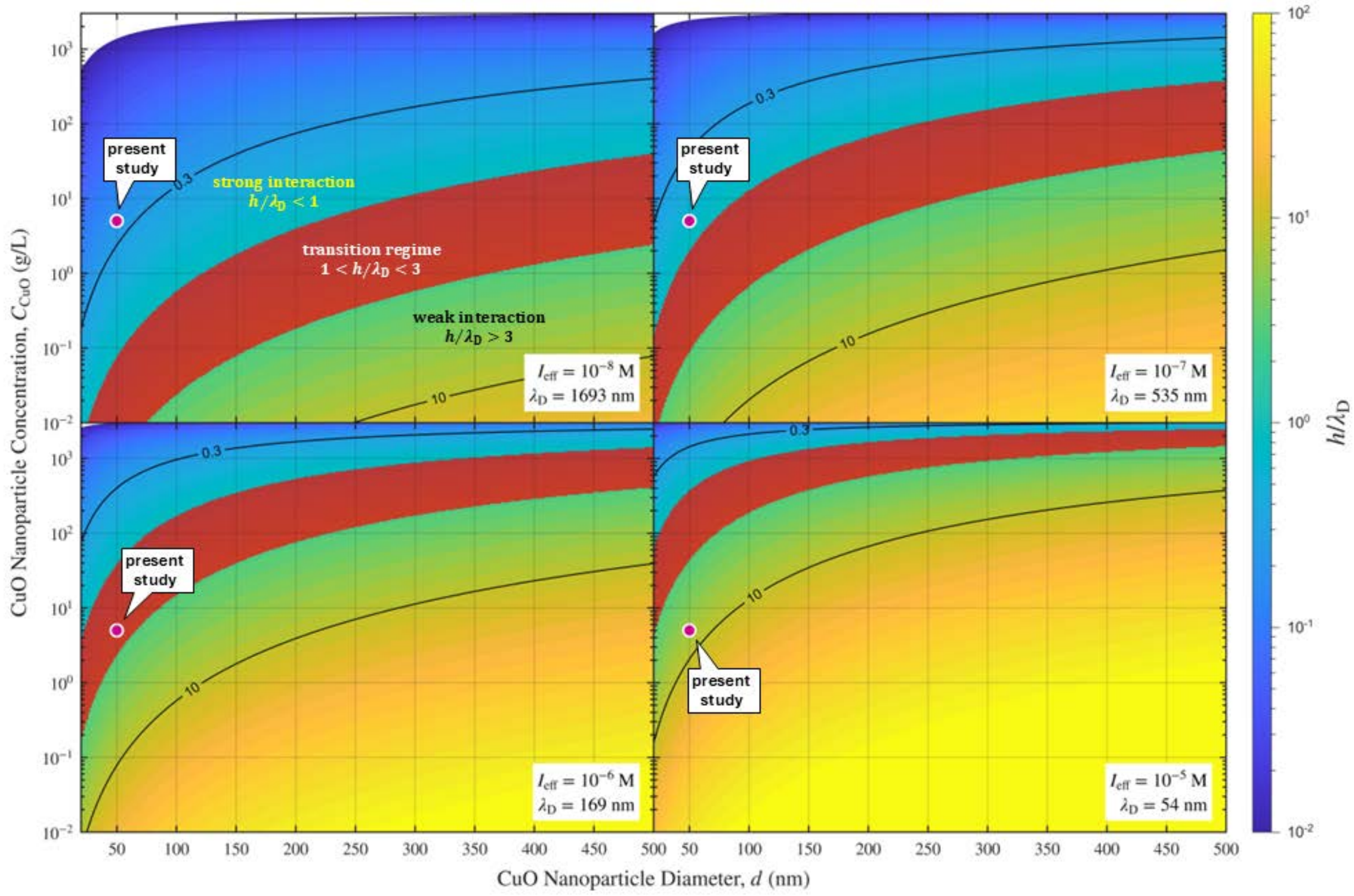

Figure S9.1 Contour plot of the double-layer overlap, h/λ_D, in dependence of the CuO nanoparticle diameter and the CuO NP concentration, for CuO NPs dispersed in absolute ethanol at room temperature. Here, h is the average distance between neighbouring nanoparticles in solution, and λ_D is the Debye length. Each panel corresponds to a different effective ionic strength of the ethanol solution, $I_{eff} = 10^{-8}$ to 10^{-5} mol/L. The red shaded band indicates the transition regime where $1 < h/\lambda_D < 3$, meaning that the electrostatic double layers are comparable to the interparticle spacing and therefore partially overlap. The marker indicates the experimental condition for spherical CuO nanoparticles with an average diameter of 50 nm dispersed in ethanol at 5 g/L, using $\varepsilon_r \approx 24.3$ and $T = 298.15$ K.

Since all nanopowders were successively washed with ethanol and water, the majority of ionic surface contamination is already removed prior to dispersing the as-washed or surface treated NPs in ethanol for the EPD process. In this case, the relative permittivity and effective ionic strength of absolute ethanol can be approximated as $\varepsilon_r \approx 24.3$ and $I_{eff} \sim 10^{-7}$ mol/L, respectively. Under these conditions, the calculated Debye length is $\lambda_D = 535$ nm, while the estimated average interparticle distance is $h = 386$ nm. Thus,

$$\frac{h}{\lambda_D} = \frac{386}{535} \approx 0.7. \tag{S2}$$

Since $h/\lambda_D < 1$, the electrostatic double layers are expected to strongly overlap under the

experimental EPD conditions. For $1 < h/\lambda_D < 3$, partial double-layer overlap occurs, whereas for $h/\lambda_D > 3$, particle–particle electrostatic interactions become much weaker and the measured ζ-potential is expected be approximately independent on the NP concentration. Hence, at CuO concentrations as high as 5 g/L, CuO NPs in ethanol are unlikely to be electrostatically isolated unless the effective ionic strength becomes sufficiently high. Consequently, ζ-potential values obtained from highly diluted CuO dispersions will not be representative of the electrokinetic behavior in the highly loaded dispersions used for EPD.

References

[1] Anastasia S. Batenkova et al. "Tuning the Surface State and Zeta Potential of Aged CuO Nanoparticles by Additive-Free Surface Treatments as Guided by XPS". In: *Adv. Mater.* (Oct. 2025). ISSN: 1521-4095. DOI: 10.1002/adma.202513030.

[2] Jerzy Dryzek and Paweł Horodek. "GEANT4 simulation of slow positron beam implantation profiles". In: *Nucl. Instrum. Methods Phys. Res., Sect. B* 266.18 (2008), pp. 4000–4009. ISSN: 0168-583X. DOI: https://doi.org/10.1016/j.nimb.2008.06.033. URL: https://www.sciencedirect.com/science/article/pii/S0168583X08008926.

[3] Ken Wada and Toshio Hyodo. "A simple shape-free model for pore-size estimation with positron annihilation lifetime spectroscopy". In: *J. Phys. Conf. Ser.* 443 (June 2013), p. 012003. ISSN: 1742-6596.
